# SUMOylation of AMPKα1 by PIAS4 specifically regulates mTORC1 signalling

**DOI:** 10.1038/ncomms9979

**Published:** 2015-11-30

**Authors:** Yan Yan, Saara Ollila, Iris P. L. Wong, Tea Vallenius, Jorma J. Palvimo, Kari Vaahtomeri, Tomi P. Mäkelä

**Affiliations:** 1 Research Programs Unit, Faculty of Medicine, University of Helsinki, Biomedicum Helsinki, Haartmaninkatu 8, PO Box 63, Helsinki FI-00014, Finland; 2 Institute of Biomedicine, University of Eastern Finland, Kuopio, PO Box 1627, FI-70211 Kuopio, Finland

## Abstract

AMP-activated protein kinase (AMPK) inhibits several anabolic pathways such as fatty acid and protein synthesis, and identification of AMPK substrate specificity would be useful to understand its role in particular cellular processes and develop strategies to modulate AMPK activity in a substrate-specific manner. Here we show that SUMOylation of AMPKα1 attenuates AMPK activation specifically towards mTORC1 signalling. SUMOylation is also important for rapid inactivation of AMPK, to allow prompt restoration of mTORC1 signalling. PIAS4 and its SUMO E3 ligase activity are specifically required for the AMPKα1 SUMOylation and the inhibition of AMPKα1 activity towards mTORC1 signalling. The activity of a SUMOylation-deficient AMPKα1 mutant is higher than the wild type towards mTORC1 signalling when reconstituted in AMPKα-deficient cells. PIAS4 depletion reduced growth of breast cancer cells, specifically when combined with direct AMPK activator A769662, suggesting that inhibiting AMPKα1 SUMOylation can be explored to modulate AMPK activation and thereby suppress cancer cell growth.

## Abstract (web summary)

AMPK senses cellular energy and switches off pathways involved in protein and fatty acid synthesis, but the selectivity of AMPK for different pathways is unclear. Here, the authors show that PIAS4-dependent SUMOylation and inactivation of AMPK preferentially restores activity of the mTORC1 pathway.

## Graphical abstract

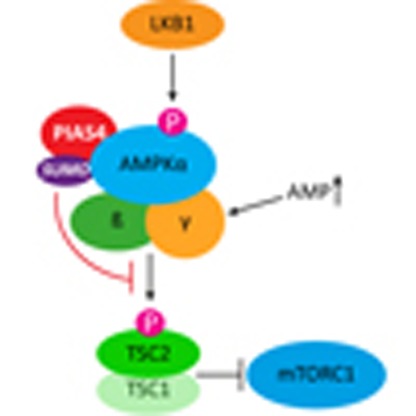

AMP-activated protein kinase (AMPK) is an evolutionarily conserved energy rheostat sensing lowered energy levels and adjusting multiple metabolic pathways to mount an appropriate reaction^1^. In general, AMPK activation stimulates ATP-producing catabolic pathways and inhibits ATP-consuming anabolic reactions by direct phosphorylation of downstream targets. Thereby, AMPK inhibits several cellular processes also important for tumour development such as fatty acid and protein synthesis^2^, and several AMPK activators including the 5-aminoimidazole-4-carboxamide-1-β-D-ribofuranoside (AICAR)^3^,^4^, metformin^5^ and A769662 (ref. 6) have been reported to inhibit cancer cell growth.

AMPK inhibits fatty acid synthesis by phosphorylating CoA carboxylase (ACC)^7^ and protein synthesis through suppression of mammalian target of rapamycin complex 1 (mTORC1)^8^. Inhibition of mTORC1 is mediated through an activating phosphorylation of tuberous sclerosis 2 (TSC2) on S1345 (ref. 9) and inhibitory phosphorylation of the regulatory-associated protein of mTOR (Raptor) on S722 and S792 (ref. 10). Although AMPK phosphorylates numerous substrates and regulates many cellular processes^2^, regulation of AMPK activity towards any one of these substrates has not been described. AMPK has also been reported to shuttle between the cytoplasm and the nucleus^11^,^12^, providing a potential mechanism for substrate selectivity.

AMPK functions as a heterotrimeric complex comprising a catalytic (AMPKα) and two regulatory subunits (AMPKβ and AMPKγ)^1^. In mammalian cells, all three subunits are encoded by more than one gene: AMPKα1 and α2; AMPKβ1 and β2; AMPKγ1, γ2 and γ3 (ref. 1). AMPKα1 and AMPKα2 demonstrate some specificity in tissue distribution^13^, subcellular localization^14^ and substrate selection^15^.

There are several ways in which mammalian AMPK is regulated. On energy deprivation, AMPK is activated in several stages^1^: increased cellular levels of AMP and ADP bind to the AMPKγ subunit, leading to stabilization of T-loop phosphorylation of AMPKα subunit provided AMPKβ is myristoylated^16^, and further allosteric activation following additional AMP binding. The mammalian AMPK complex has been reported to be inhibited by mutant p53 (ref. 17), adaptor protein α-SNAP^18^, glycogen synthase kinase 3-mediated phosphorylation of S479 inhibiting T-loop phosphorylation^19^, E3 ubiquitin ligase Wwp1 (ref. 20) or CIDE family protein Cidea^21^, and activated by scaffold protein KSR2 (ref. 22) or p53 targets Sestrin1 and Sestrin2 (ref. 23). With the exception of S479 phosphorylation^19^, the inhibitory mechanisms have not been characterized beyond protein–protein interactions with AMPK.

In budding yeast, the AMPK orthologue SNF1 is regulated by several posttranslational modifications. Acetylation of Sip2 (β-subunit) inhibits SNF1 kinase activity and prolongs lifespan^24^. SUMOylation of Snf1 (α-subunit) on a residue not conserved in mammalian AMPKα inhibits the kinase by internal SUMO-interacting motif interaction and by targeting Snf1 for degradation^25^, which possibly involves Snf1 ubiquitination^26^. Mammalian AMPKα has been reported to be ubiquitinated^27^ and targeted for degradation by ubiquitination in some cancers overexpressing the MAGE-A3/6-TRIM28 ubiquitin ligase^28^, whereas other posttranslational modifications of AMPKα have not been identified.

Here we present evidence that AMPK activation induces SUMOylation of its catalytic subunit AMPKα1. SUMO E3 ligase PIAS4 catalyses the SUMOylation of AMPKα1, which inhibits AMPK activity specifically towards mTORC1 signalling. Our results therefore uncovered a novel regulatory mechanism by which AMPKα1–mTORC1 signalling is specifically modulated.

## Results

### PIAS4 interacts with AMPK and modulates mTORC1 signalling

To identify novel interactors of AMPK, we performed yeast two-hybrid screens using human AMPKα1 and AMPKα2 GAL4–DBD fusion proteins as baits. The screens identified both well-characterized AMPKα interactors such as AMPKγ1, TRIP6 (ref. 29) and PPP1R12C^30^, as well as putative novel interactors (Supplementary Table 1) including SUMO E3 ligase PIAS3. Owing to the significant sequence homology of the four mammalian PIAS proteins (PIAS1, PIASx/PIAS2, PIAS3 and PIAS4/PIASy)^31^, all of these were included in subsequent validation. Affinity purification of the glutathione *S*-transferase (GST)-tagged AMPKα1 or AMPKα2 from HEK293 cells followed by western blotting (WB) indicated that both PIAS3 and PIAS4 associated with AMPKα1 and AMPKα2 (Supplementary Fig. 1a), whereas PIAS1 or PIASx were not detected. In reciprocal experiments, affinity purification of GST–PIAS3 or GST–PIAS4 demonstrated co-purification of all three endogenous AMPK subunits (Supplementary Fig. 1b).

**Figure 1:**
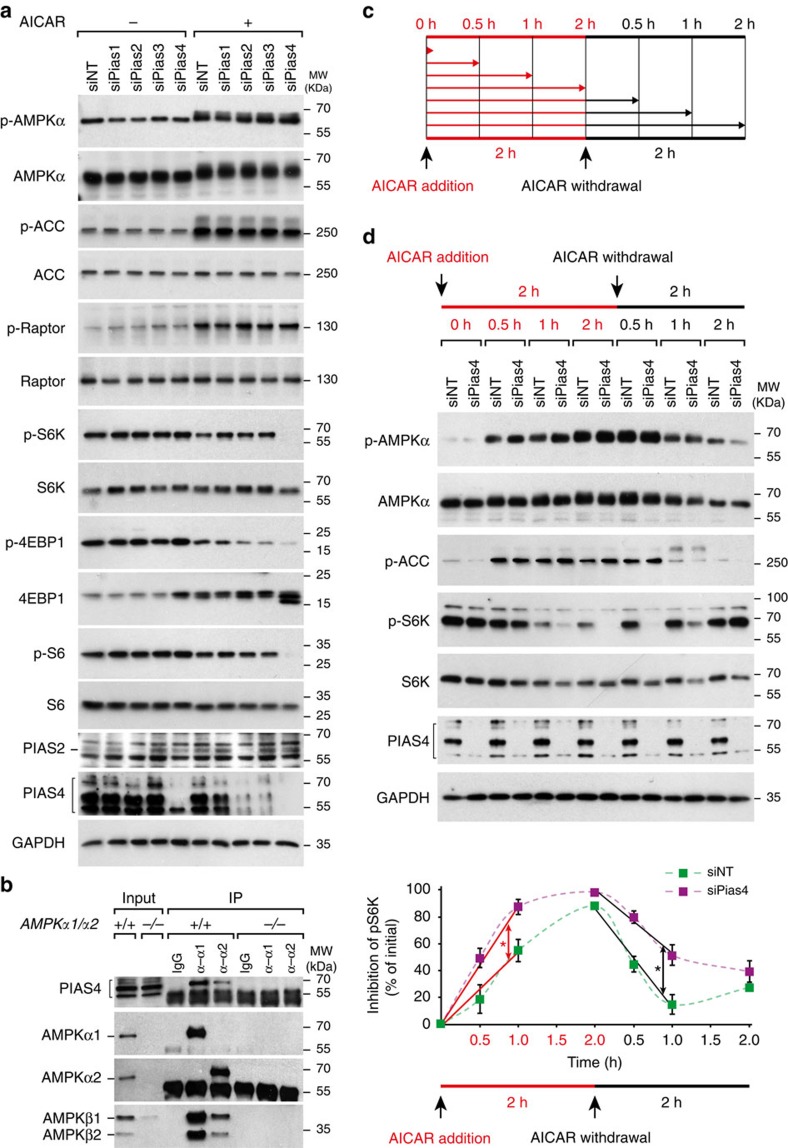
PIAS4 interacts with AMPK and modulates mTORC1 signalling. (**a**) Cell lysates from immortalized MEFs transfected with indicated siRNA pools (NT, non-targeting) and 72 h later treated with vehicle (−) or 2 mM AICAR (+) for 2 h were analysed by SDS–PAGE and WB using indicated antibodies. Brackets in PIAS4 blot denotes specific signal. (**b**) Lysates from AMPK*α1*^*+/−*^*;α2*^*+/+*^ or AMPK*α1*^−/−^*;α2*^−/−^ immortalized MEFs were analysed directly (Input) or following IP using control IgG or antibodies against AMPKα1 (α-α1) or AMPKα2 (α-α2) and protein G-Sepharose by WB analysis with the antibodies. The bracket of PIAS4 blot denotes specific signals. (**c**) A scheme of the AICAR addition and withdrawal. Immortalized MEFs were analysed either at indicated times (red arrows) after AICAR treatment (AICAR addition) or at indicated times (small black arrows) following AICAR washout by PBS and changing into media without AICAR (AICAR withdrawal). (**d**) Upper panels: immortalized MEFs transfected with siNT or siPias4 and 72 h later were subjected for AICAR addition, and withdrawal as depicted in **c** and analysed as in **a**. The bracket of PIAS4 blot denotes specific signals. Lower panels: p-S6K/S6K levels from each time points were quantified (mean±s.e.m., *n*=3) and p-S6K inhibition (%) is shown as relative to initial time point (green dotted line: siNT; purple dotted line: siPias4). The speed of p-S6K inhibition after AICAR addition and p-S6K restoration following AICAR withdrawal were calculated from the slopes of least-squares fit to linear regressions of p-S6K inhibition levels versus the first three time points after AICAR addition (0, 0.5 and 1 h) (red continuous lines; siNT slope=0.551 and siPias4 slope=0.875; *P<0.05) and AICAR withdrawal (0, 0.5 and 1 h) (black continuous lines; siNT slope=−0.730 and siPias4 slope=−0.469; *P<0.05), respectively (analysis of variance followed by Fisher's least significant difference test). Representative figures from two (**a**,**b**) or three (**c**) experiments are shown.

To investigate whether PIAS E3 ligases regulate AMPK signalling, we analysed AMPK activation on AICAR^32^ treatment following small interfering RNA (siRNA)-mediated downregulation of Pias1–4 in immortalized mouse embryonic fibroblasts (MEFs; Fig. 1a and Supplementary Fig. 2a). In knockdown controls, by WB PIAS4 was detected as several bands and are indicated as brackets. It was also noted that Pias2 and Pias3 knockdown concomitantly reduced PIAS4 levels to some extent following AICAR treatment, consistent with the observation that Pias4 messenger RNA level was reduced in the testis of Pias2^−/−^ mice^33^. Depletion of Pias1, Pias2 and also Pias3 using pooled siRNAs did not result in any noticeable effects (Fig. 1a). By contrast, depletion of Pias4 enhanced suppression of mTORC1 signalling reflected by enhanced dephosphorylation of mTORC1 substrates p70 S6 kinase (S6K), eIF4E binding protein 1 (4EBP1) and ribosomal protein S6 (S6). The result was verified by quantification of p-S6K/S6K (Supplementary Fig. 2b) and by using two independent siRNAs against Pias4 (Supplementary Fig. 2c). Effects of Pias4 depletion were noted at varying AICAR concentrations: the maximal suppression of mTORC1 in control cells was already reached with 0.5 mM AICAR, whereas with the same concentration in Pias4-depleted cells the ability of AICAR in suppressing mTORC1 was higher and the suppression of mTORC1 was further increased up to 2 mM AICAR (Supplementary Fig. 2d). The result suggests that a larger pool of AMPKα is available for AICAR activation in Pias4-depleted cells. The enhanced AMPK signalling following Pias4 depletion towards mTORC1 was not associated with changes in AMPK activation as measured by phosphorylation of AMPKα-T172 (Fig. 1a; p-AMPKα; phosphorylation associated with slightly slower migration of AMPKα in these conditions) or by changes in AMPK signalling towards ACC and Raptor (Fig. 1a; p-ACC and p-Raptor).

Unlike AICAR treatment, Pias4 knockdown had no effects on mTORC1 signalling in the cells treated with unspecific AMPK activator metformin or phenformin (Supplementary Fig. 3), antidiabetic biguanides that suppress mTORC1 signalling independent of AMPK and TSC2 (ref. 34). Importantly, by using wild-type (WT; *AMPKα1*^*+/−*^*;α2*^*+/+*^) or AMPKα-null (*AMPKα1*^−/−^*;α2*^−/−^) immortalized MEFs and validated AMPKα1- and AMPKα2-specific antibodies (Supplementary Fig. 1c,d), we found that endogenous PIAS4 co-immunoprecipitates with both AMPKα1 and AMPKα2 (Fig. 1b). These results indicate that PIAS4 associates with AMPK and inhibits AMPK activity specifically towards mTORC1 signalling.

In yeast, the E3 ligase Mms21 is required for glucose-induced inactivation of Snf1 and degradation of sensor/receptor-repressor (SRR) pathway component Mth1 (ref. 25). We therefore investigated whether AMPK inactivation following removal of AICAR is regulated by PIAS4 in mammalian cells by analysing the phosphorylation state of AMPKα, ACC and S6K (Fig. 1d) after indicated times following addition (AICAR addition) and following withdrawal of AICAR (AICAR withdrawal) as depicted in Fig. 1c. Based on the phosphorylation state of AMPKα, ACC and S6K (Fig. 1d), Pias4 depletion led to a significantly faster inhibition of p-S6K following AICAR addition and delayed restoration of p-S6K following AICAR withdrawal (Fig. 1d, AICAR withdrawal), indicating that PIAS4 is essential for both activation and inactivation of AMPK and suggesting conservation in the manner SUMO E3 ligase regulates the inactivation of AMPK/SNF1 kinase.

### PIAS4 modulates mTORC1 signalling via AMPKα1 and TSC2

**Figure 2:**
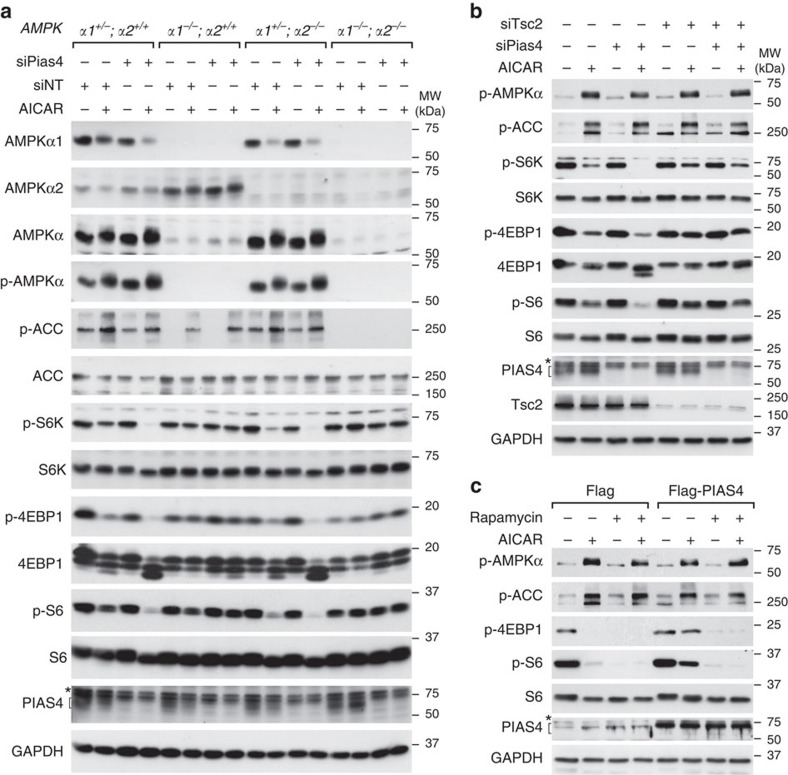
PIAS4 modulates mTORC1 signalling via AMPKα1 and TSC2. (**a**) Immortalized MEFs with variable *AMPKα* genotypes as indicated on the top were transfected with siNT or siPias4 and 72 h later treated with vehicle (−) or 2 mM AICAR (+) for 2 h. Cell lysates were analysed by SDS–PAGE and WB using antibodies indicated on the left. For PIAS4 blot, asterisk denotes unspecific signals and the bracket denotes specific signals. (**b**) Immortalized MEFs transfected with siPias4 and siTsc2 as indicated were treated 72 h later with vehicle (−) or 2 mM AICAR (+) for 2 h and analysed as in **a**. For PIAS4 blot, the asterisk denotes unspecific signals and the bracket denotes specific signals. (**c**) NIH3T3 cells were transfected with vector control (Flag) or Flag–PIAS4 plasmids and 24 h later treated with vehicle, 2 mM AICAR, 50 nM rapamycin or both for 2 h and analysed as in **a**. For PIAS4 blot, the asterisk denotes unspecific signals and the bracket denotes specific signals. Representative figures from two experiments (**a**–**c**) are shown.

To directly address the roles of AMPKα1 and AMPKα2 in the regulation of mTORC1 substrates and modulation by PIAS4, we used immortalized MEFs with WT AMPKα (*α1*^*+/−*^*;α2*^*+/+*^) or with deletions of AMPKα1 (*α1*^−/−^*;α2*^*+/+*^), AMPKα2 (*α1*^*+/−*^*;α2*^−/−^) or both (*α1*^−/−^*;α2*^−/−^). In control MEFs, AICAR treatment lead to the activation of AMPKα noted in expected changes in mTORC1 substrates (p-S6K, p-S6 and p-4EBP1) and p-ACC, and Pias4 depletion specifically potentiated mTORC1 suppression by AICAR (Fig. 2a, α1^+/−^;α2^*+/+*^, PIAS4 levels following Pias4 knockdown reduced by 69% in *α1*^*+/−*^*;α2*^*+/+*^ cells in –AICAR and +AICAR samples Supplementary Fig. 4a). Interestingly, in MEFs containing only AMPKα2 (*α1*^−/−^*;α2*^*+/+*^), AICAR treatment did not alter mTORC1 signalling (Fig. 2a and Supplementary Fig. 4a), whereas ACC was still activated; Pias4 depletion did not have any effects (Fig. 2a, PIAS4 levels reduced by 57% in –AICAR condition and 52% in +AICAR samples; Supplementary Fig. 4a). Similar to control MEFs, in MEFs containing only AMPKα1 (*α1*^*+/−*^*;α2*^−/−^), AICAR treatment efficiently inhibited mTORC1 signalling and activated ACC similar to control MEFs; Pias4 depletion specifically potentiated the inhibition of mTORC1 signalling by AICAR (Fig. 2a, PIAS4 levels reduced by 68% in –AICAR condition and 62% in +AICAR condition; Supplementary Fig. 4a). As expected, in MEFs lacking both AMPKα1 and AMPKα2 (*α1*^−/−^*;α2*^−/−^), phosphorylation of ACC was abolished and AICAR treatment did not suppress mTORC1 signalling; Pias4 depletion no longer have any effects on mTORC1 signalling (Fig. 2a, PIAS4 levels reduced by 68% in –AICAR condition and 78% in +AICAR condition; Supplementary Fig. 4a). Depletion of AMPKβ2 did not detectably affect PIAS4-mediated mTORC1 activation (Supplementary Fig. 4b), although it was reported that AMPKβ2 is modified by PIAS4-dependent SUMOylation^35^.

AMPK suppresses mTORC1 signalling through two pathways: TSC2 or Raptor phosphorylation. As PIAS4 depletion did not affect Raptor phosphorylation (Fig. 1a), we subsequently examined the requirement of TSC2 in PIAS4-regulated mTORC1 activity. To this end, we depleted both PIAS4 and TSC2 in immortalized MEFs. Again, Pias4 depletion potently enhanced the inhibition of mTORC1 following AICAR treatment in control MEFs as seen, for example, by p-S6K (Fig. 2b and Supplementary Fig. 4c, −siTsc2). This effect was almost completely blocked following TSC2 depletion (Fig. 2b and Supplementary Fig. 4c, +siTsc2), demonstrating that TSC2 is required for mTORC1 inhibition on PIAS4 depletion.

mTORC1 integrates signals from growth factors, amino acids, stress and energy levels, to regulate protein synthesis and cell growth^36^, and its activity can be directly inhibited by rapamycin^37^. To determine that dephosphorylation of mTORC1 substrates in PIAS4-depleted cells was not due to direct activation of mTORC1 by PIAS4, we treated cells with vehicle, 2 mM AICAR, 50 nM rapamycin or both for 2 h following transfection with vector (Flag) or Flag–PIAS4. AICAR-induced mTORC1 inhibition was impaired by Flag–PIAS4 overexpression, whereas rapamycin eliminated the mTORC1 activity in both Flag- and Flag–PIAS4-transfected cells (Fig. 2c and Supplementary Fig. 4d). Collectively, these data indicate that PIAS4 modulates AMPKα1-mediated inhibition of mTORC1 signalling via TSC2.

### PIAS4 inhibits AMPKα1 activity towards TSC2

**Figure 3:**
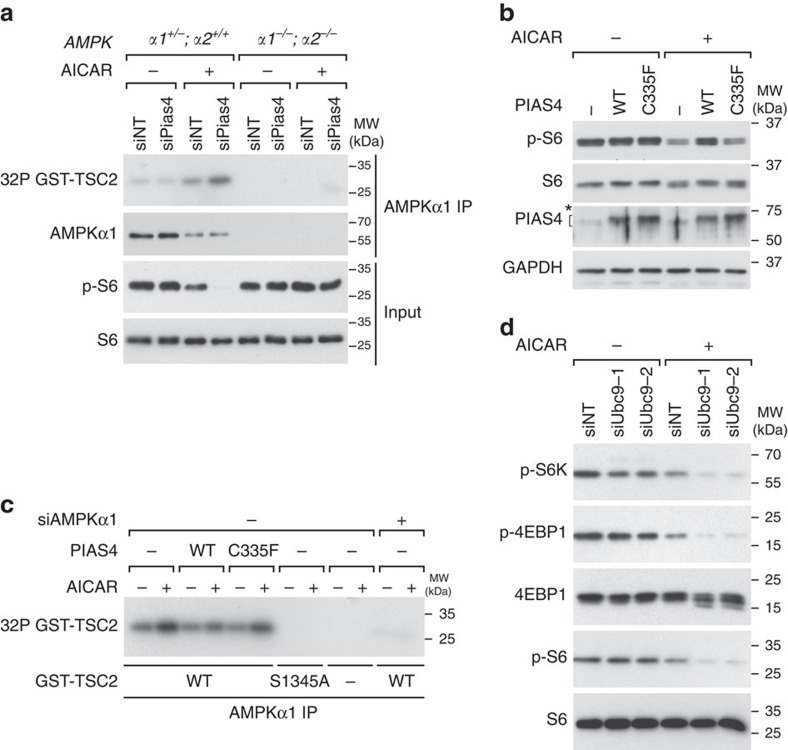
PIAS4 inhibits AMPKα1 activity in a SUMO E3 ligase-dependent manner. (**a**) AMPKα1 immunoprecipitates from cells with the indicated *AMPKα* genotypes transfected with indicated siRNAs followed by treatment with vehicle (−) or AICAR (+) were used to phosphorylate recombinant GST–TSC2 (1,300–1,367) *in vitro* in the presence of [γ-^32^P] ATP followed by SDS–PAGE and autoradiography (32P GST–TSC2) or WB analysis with antibodies as indicated on the left. (**b**) NIH3T3 cells transfected with vector control (−), Flag–PIAS4-WT (WT) or Flag–PIAS4-C335F (C335F) plasmids and 24 h later treated with vehicle (−) or 2 mM AICAR (+) for 2 h were lysed and analysed by SDS–PAGE and WB with antibodies indicated on the left. For PIAS4 blot, the asterisk denotes unspecific signals and the bracket denotes specific signals. (**c**) AMPKα1 immunoprecipitates from NIH3T3 cells transfected with non-targeting (−) or AMPKα1 (+) siRNAs and PIAS4 plasmids as indicated, and treated for 2 h with vehicle (−) or 2 mM AICAR (+) were used in kinase assays as in **a** with recombinant GST–TSC2 (1,300–1,367) control (WT) or S1345A mutant as substrates. (**d**) Lysates from immortalized MEFs transfected with the non-targeting (siNT) siRNA pool or two independent siRNAs against mouse Ubc9 (siUbc9-1 and siUbc9-2) and 72 h later treated with vehicle (−) or 2 mM AICAR (+) for 2 h were analysed by WB with indicated antibodies. Representative figures from two (**b**,**d**) or three (**a**,**c**) experiments are shown.

We next investigated whether inhibition of mTORC1 following PIAS4 depletion is due to enhanced kinase activity of AMPKα1 towards TSC2. To this end, we compared the ability of immunoprecipitated AMPKα1 from control (siNT) or PIAS4-depleted (siPias4) MEFs, to phosphorylate a recombinant GST–TSC2 fragment (1,300–1,367) containing the AMPK phosphorylation site S1345 (ref. 9). As expected, no AMPKα1 kinase activity was detected in precipitates from *AMPKα1*^−/−^*;α2*^−/−^ MEFs (Fig. 3a and Supplementary Fig. 5a). Notably, AMPKα1 activity was significantly higher in AICAR-treated lysates from Pias4-depleted cells (Fig. 3a and Supplementary Fig. 5a). This result suggests PIAS4 inhibits AMPKα1 activity towards TSC2. The specificity of Pias4 inhibition noted in cells could not be recapitulated in the reconstituted system using the SAMS peptide as a surrogate for ACC^38^ (Supplementary Fig. 5b).

To determine whether PIAS4 SUMO E3 ligase activity is required to regulate AMPKα1, we mutated PIAS4-C335 to phenylalanine (C335F), to disrupt the E3 ligase activity^39^. Unlike PIAS4-WT, the E3 ligase-deficient PIAS4 C335F (PIAS4-C335F) did not inhibit AMPK as determined by unaffected dephosphorylation of S6 (Fig. 3b and Supplementary Fig. 5c). We next examined the *in vitro* phosphorylation of GST–TSC2 by AMPKα1 immunoprecipitated from Flag–PIAS4-WT- or Flag–PIAS4-C335F-expressing cells, and noted that the ability of PIAS4 to inhibit AMPKα1 kinase activity was significantly reduced by the C335F mutation (C335F) (Fig. 3c and Supplementary Fig. 5d). These results indicate PIAS4 inhibits AMPKα1 through its SUMO E3 ligase activity.

To further explore the possible role of SUMOylation in the regulation of mTORC1, we depleted Ubc9, the sole SUMO E2 enzyme^31^, with two independent siRNAs (siUbc9-1 and siUbc9-2) in immortalized MEFs. Depletion of Ubc9 enhanced AICAR-induced suppression of mTORC1 signalling (Fig. 3d and Supplementary Fig. 5e), suggesting that SUMOylation is involved in inhibiting AMPKα1 activity towards TSC2.

### AMPKα1 and AMPKα2 are SUMOylated by PIAS4

**Figure 4:**
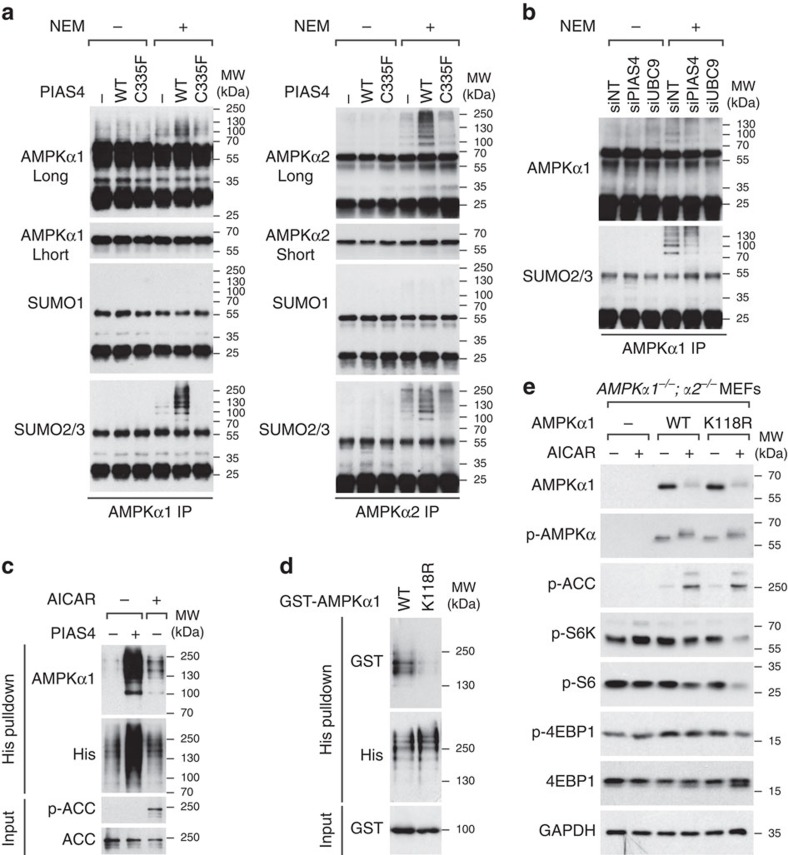
AMPKα1 and AMPKα2 are SUMOylated by PIAS4. (**a**) HEK293 cells transfected with vector control (−), Flag–PIAS4-WT (WT) or Flag–PIAS4-C335F (C335F) plasmids were lysed 24 h later with or without 20 mM deSUMOylation inhibitor NEM followed by anti-AMPKα1 (left panels) or anti-AMPKα2 (right panels) IP and WB analysis with indicated antibodies. (**b**) AMPKα1 immunoprecipitates prepared as in **a** from HEK293 cells transfected with non-targeting (siNT), PIAS4 (siPIAS4) or UBC9 (siUBC9) siRNA pools were analysed by WB with anti-AMPKα1 or anti-SUMO2/3 antibodies as indicated. (**c**) Upper panels: WB analysis using AMPKα1 or poly-histidine (His) antibodies from metal-affinity chromatography purifications (His pulldown) prepared from denaturing lysates of HEK293 cells co-transfected with plasmids encoding 6His–SUMO3 and either vector control (−) or Flag–PIAS4 (+) and 24 h later treated with vehicle (−) or 2 mM AICAR (+) for 4 h. Lower panels: WB analysis of total lysates (Input) with the indicated antibodies. (**d**) Upper panels: WB analysis using anti-GST or poly-histidine (His) antibodies from His pulldown prepared from denaturing lysates of HEK293 cells co-transfected with plasmids encoding 6His–SUMO3 and Flag–PIAS4, and either WT or K118R form of GST–AMPKα1. Lower panel: WB analysis of total lysates (Input) with the indicated antibodies. (**e**) Immortalized *AMPKα1*^−/−^*;α2*^−/−^ MEFs transduced with lentivirus expressing control vector (−) or AMPKα1-WT (WT), or AMPKα1-K118R (K118R) and selected with blasticidin (10 μg ml^−1^) for 1 week were treated with vehicle (−) or 2 mM AICAR (+) for 2 h. Cell lysates were analysed by SDS–PAGE and WB using antibodies indicated on the left. Representative figures from two (**a**–**c**) or three (**d**,**e**) experiments are shown.

Considering the requirement of the PIAS4 E3 ligase activity for AMPK inhibition, it was of interest to directly investigate whether AMPKα is SUMOylated. To this end, AMPKα1 (Fig. 4a, left panels) or AMPKα2 (Fig. 4a, left panels) were immunoprecipitated in the absence or presence of deSUMOylation inhibitor N-ethylmaleimide (NEM) from HEK293 cells transfected with vector control (−), Flag–PIAS4-WT (WT) or Flag–PIAS4-C335F (C335F). Subsequent WB analysis demonstrated the presence of SUMO2/3- but not SUMO1-modified forms of both AMPKα1 and AMPKα2 in the NEM-treated lysates of control cells (Fig. 4a, lanes NEM+ PIAS4−). The amount of SUMOylated AMPKα1 and AMPKα2 was significantly increased following PIAS4-WT but not C335F overexpression (Fig. 4a, lanes NEM+ PIAS4+), consistent with earlier results demonstrating PIAS4 E3 ligase activity is required for inhibition of AMPK (Fig. 3b, Supplementary Fig. 5c, Fig. 3c and Supplementary Fig. 5d). Subsequent 6His–SUMO purifications confirmed that both SUMO2 and SUMO3 can be used to modify both AMPKα1 and AMPKα2 (Supplementary Fig. 6a), yet distinct patterns of SUMOylated AMPKα1 and AMPKα2 induced by PIAS4 were noticed when analysing the SUMOylation of GST–AMPKα1 and GST–AMPKα2 following nickel affinity purification of 6His–SUMO3 (Supplementary Fig. 6b).

Given the requirement of AMPKα1 in mediating the effect of PIAS4 on mTORC1 signalling (Fig. 2a), we chose to further analyse AMPKα1 SUMOylation in more detail. Knockdown of UBC9 (siUBC9) or PIAS4 (siPIAS4) inhibited SUMOylation of AMPKα1 compared with siNT (Fig. 4b, lanes NEM+). Following nickel affinity purification of 6His–SUMO3, SUMOylated AMPKα1 in PIAS4 untransfected cells was greatly enhanced following AICAR treatment (Fig. 4c, lane AICAR+). The results suggest that PIAS4 is required for the SUMOylation of AMPKα1 in cells. We subsequently attempted to reconstitute SUMOylation of AMPKα1 *in vitro* using SUMO3/E1/E2 and bacterially produced AMPK complex (α1, β1 and γ1) and GST–PIAS4. As a positive control for the assay, SUMOylation of p53 was detected in the presence of SUMO3/E1/E2/ATP and its SUMOylation was markedly enhanced by GST–PIAS4 (Supplementary Fig. 6c, right panels), confirming the previous report^39^. In the same setting, several slower migrating bands were detected with AMPKα1 antibody in the presence of SUMO3/E1/E2/ATP and addition of GST–PIAS4 led to an increase of high-molecular-weight bands (Supplementary Fig. 6c, left panels, bracket). The results demonstrate that AMPKα1 can be SUMOylated in a PIAS4-dependent manner.

The most probably predicted SUMOylation sites on AMPKα1 are lysines in the context of LK^152^PE and FK^280^QD. However, mutating either lysine to arginine did not noticeably affect the SUMOylation of AMPKα1 (Supplementary Fig. 6d, K152R and K280R). Subsequent mutagenesis and analysis of all remaining lysines in AMPKα1 revealed that substitution of lysine 118 to arginine greatly attenuated the SUMOylation of AMPKα1 (Fig. 4d, K118R), suggesting K118 represents a major SUMOylation site on AMPKα1.

Our earlier results provide evidence that SUMOylation is involved in inhibiting AMPKα1 activity towards TSC2 and thus predict that the SUMOylation-deficient mutant AMPKα1-K118R would be resistant to this inhibition. To test this we reconstituted AMPKα-null MEFs with AMPKα1-WT or AMPKα1-K118R and analysed their ability to suppress mTORC1 signalling following AMPK activation. The results indicate that AMPKα1-K118R reconstituted cells demonstrate an elevated ability to suppress mTORC1 signalling (Fig. 4e and Supplementary Fig. 6e), whereas the activity towards ACC was comparable to the WT control (Fig. 4e, p-ACC). The results provide further evidence that SUMOylation of AMPKα1 specifically regulates mTORC1 signalling.

To investigate whether SUMOylation affects AMPKα1 subcellular localization, we initially analysed localization of total AMPKα1 from AICAR-activated AMPKα-null MEFs reconstituted with GST–AMPKα1-WT or GST–AMPKα1-K118R, which both form complexes with AMPKβ1/2 in both cytoplasmic and nuclear compartment (Supplementary Fig. 7c). Considering the low stoichiometry of SUMOylated AMPKα1 (Fig. 4a, <5%), the nuclear/cytoplasmic distribution was quantified from >1,500 AMPKα1-WT and AMPKα1-K118R cells (see Methods), and demonstrated a 2.2% enrichment of total AMPKα1-K118R in the cytoplasm compared with AMPKα1-WT (Supplementary Fig. 7a, 60.1% versus 57.9%, *P*<0.05, Student's *t*-test).

We subsequently investigated in which subcellular compartment AMPKα1 SUMOylation occurs using a proximity ligation assay (PLA)^40^ in AMPKα-null MEFs reconstituted with WT or SUMOylation-deficient GST–AMPKα1. Although no detectable PLA signal was noted from control MEFs, cells expressing WT AMPKα1 (AMPKα1-WT) demonstrated a strong and predominantly nuclear (83.4%) PLA signal (Supplementary Fig. 7b). PLA signal in the AMPKα1-K118R was dramatically reduced to 3% of that in AMPKα1-WT cells consistent with the earlier results, indicating K118 represents a major SUMOylation site. In summary, the localization analyses indicate that SUMOylated AMPKα1 is mostly nuclear, which could be due, for example to reduced nuclear–cytoplasmic shuttling^11^,^12^.

### PIAS4 depletion reduces breast cancer cell proliferation

**Figure 5:**
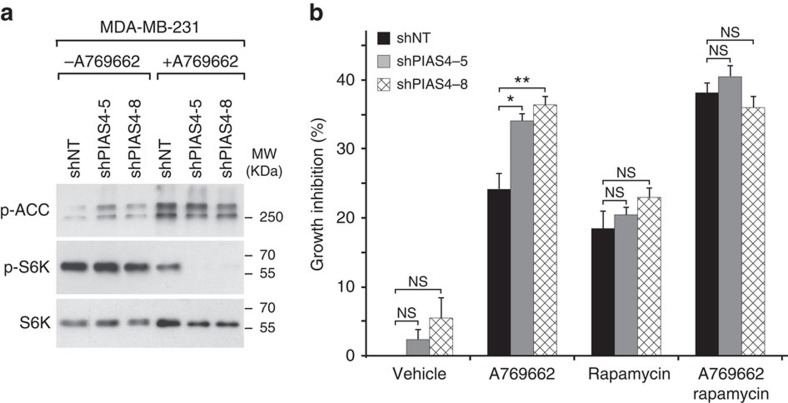
PIAS4 depletion potentiates AMPK activity towards mTORC1 and inhibits proliferation of MDA-MD-231 breast cancer cells. (**a**) WB analysis with p-ACC, p-S6K and S6K antibodies of lysates from control (shNT) or PIAS4-depleted (shPIAS4-5 and shPIAS4-8) MDA-MB-231 breast cancer cells treated with vehicle (−) or 150 mM A769662 (+) for 12 h. (**b**) Growth inhibition analysis of control (shNT) and PIAS4-depleted (shPIAS4-5 and shPIAS4-8) MDA-MB-231 breast cancer cells treated with vehicle, A769662 (150 μM), rapamycin (10 μM) or both as described in Methods. Cell proliferation was assessed by comparing number of viable cells on day 4 with those of day 1 and the growth inhibition (%)was determined by calculating the percentage of cell proliferation reduction compared that with the control (shNT+vehicle). Data are means+s.e.m. (*n*=3); significances of differences by Student's *t*-test are indicated (**P<0.01; *P<0.05; NS, *P*>0.05). Representative figures from two (**a**) or three (**b**) experiments are shown.

As PIAS4 depletion was found to potentiate AMPK activity towards mTORC1 (Fig. 1a) and AMPK activation can inhibit cancer cell growth, we next investigated whether PIAS4 depletion affect cancer cell growth in MDA-MB-231 breast cancer cells reported to be sensitive to AMPK activation^4^,^41^. To this end, we depleted PIAS4 using lentiviral transduction of two independent short hairpin RNAs (Supplementary Fig. 8, shPIAS4-5 or shPIAS4-8) and thereafter activated AMPK using A769662 (ref. 42), which stabilizes the interaction between AMPKα and AMPKβ subunit^43^, and is independent of AMPKα T-loop phosphorylation^44^. As expected, administration of A769662 for 12 h led to phosphorylation of ACC and dephosphorylation of mTORC1 substrate S6K (Fig. 5a). In A769662-treated cells, PIAS4 depletion markedly enhanced the dephosphorylation of S6K without altering ACC phosphorylation (Fig. 5a), concordant with observations in MEFs following AICAR treatment (Fig. 1a). Next, we analysed proliferation of control (shNontargeting clone (shNT)) or PIAS4-depleted (shPIAS4-5 or shPIAS-8) MDA-MB-231 cells treated with A769662, rapamycin or both for 72 h. As previously reported, both A769662 (ref. 41) and rapamycin^45^ treatment alone modestly suppressed cell growth in control shNT cells. A769662 and rapamycin together had additive effects (Fig. 5b), suggesting that AMPK activation suppress growth partly through inhibition of lipogenesis as noted in glioblastoma cells^46^. Importantly, PIAS4 downregulation specifically potentiated the A769662-induced inhibition of proliferation, whereas no significant changes were noted in vehicle control or A769662+rapamycin-treated cells (Fig. 5b). These results indicate PIAS4 depletion enhances A769662-mediated inhibition of proliferation of MDA-MB-231 breast cancer cells and this occurs via mTORC1 inhibition.

## Discussion

Our study identifies PIAS4-mediated SUMOylation as a novel and specific regulatory mechanism to attenuate AMPKα1 activity towards mTORC1 following AMPK activation.

PIAS4 was also noted to be important for rapid inactivation of AMPK following AICAR withdrawal (Fig. 1d). Although AMPK inactivation and its regulation has not been studied in mammalian cells, our observation is consistent with results obtained in budding yeast, where SUMO E3 ligase Mms21 was required for Snf1 inactivation^25^, suggesting that SUMOylation is involved in both Snf1 and AMPKα inactivation. The regulation of SUMOylation however appears to differ between yeast and mammalian cells, as in yeast SUMOylation occurs during Snf1 inactivation and is associated with non-phosphorylated Snf1 (ref. 25), whereas in mammalian cells SUMOylation of AMPKα1 was low when AMPK was inactive (Fig. 4c, lane AICAR− PIAS4−) and induced concomitant with AMPK activation. Another difference between yeast and mammalian SUMOylation is that, in yeast SUMOylation leads to Snf1 degradation^25^, whereas stability of AMPKα1 was not detectably altered following PIAS4 depletion (Fig. 3a) or when comparing WT and the SUMOylation-deficient mutant in reconstituted AMPKα-knockout MEFs (Fig. 4e).

The availability of MEFs deficient for AMPKα1, AMPKα2 or both allowed robust analysis of isoform specificity. Interestingly, the ability of AMPK to regulate mTORC1 signalling was lost in cells only containing AMPKα2 (Fig. 2a and Supplementary Fig. 4a), suggesting that TSC2 phosphorylation *in vivo* is specifically mediated by AMPKα1 in MEFs, and providing an explanation for the inability of PIAS4 to regulate mTORC1 signalling in these cells. An alternative explanation could be provided by the significantly lower levels of total AMPKα in MEFs only expressing AMPKα2 (Fig. 2a) and different sensitivities of AMPK substrates as proposed by Houde *et al*.^47^ In that case, TSC2 phosphorylation would require significantly higher levels of AMPK activity compared with phosphorylation of ACC, which was still detected in cells with only AMPKα2 following AICAR treatment (Fig. 2a).

Activation of AMPK is associated with nuclear translocation^11^,^48^, providing a mechanism by which the predominantly nuclear PIAS4 is associated with AMPKα1 and consistent with our observation that SUMOylated AMPKα1 was detected primarily in the nucleus (Supplementary Fig. 7b).

The apparent low (<5%) stoichiometry of SUMOylation of AMPKα1 (Fig. 4a,b) together with the 5- to 20-fold activation of AMPK towards mTORC1 in conditions inhibiting SUMOylation suggests SUMOylation represents a licensing event necessary for a subsequent, more stable modification as described for NEMO/IKKγ^49^ and SF-1 (ref. 50).

The nuclear SUMOylation of AMPKα1 and cytoplasmic localization of TSC2 suggests that TSC2 phosphorylation is mediated by AMPKα1 that has shuttled from the nucleus to the cytoplasm following SUMOylation and licensing. Such a requirement could provide the specificity observed between AMPKα1 substrates TSC2 and ACC/Raptor, where AMPKα1 phosphorylation of the latter substrates would not require prior shuttling. Although further investigations are required to dissect these mechanisms, our observation that AMPK activity is regulated in a substrate-specific manner is interesting, considering the various AMPK modifications and interacting proteins.

AMPK is physiologically activated by AMP/ADP via three mechanisms: inhibition of α-Thr172 dephosphorylation by both AMP and ADP, promotion of α-Thr172 phosphorylation and allosteric activation of already phosphorylated AMPK by AMP^51^. AMPK can be also activated by A769662 independently of AMP/ADP and AMPKα T-loop phosphorylation^44^ through stabilization of the interaction between AMPKα and AMPKβ subunits^43^. Here, PIAS4 depletion enhanced the ability of both AICAR and A769662, to suppress mTORC1 signalling without altering AMPKα protein levels or α-Thr172 phosphorylation (Figs 1a, 3a and 4e), suggesting that SUMOylation regulates AMPKα1 activity at the level of allosteric activation, but in a more stable manner based on the maintenance of this regulation following immunoprecipitation (IP). Thus inhibition of SUMOylation could be used to further potentiate AMPK activation following increased AMP/ADP level or the stabilization of AMPKα/AMPKβ interaction by A769662.

Enhancement of AMPK activity towards mTORC1 and associated cytostatic effects in MDA-MB-231 breast cancer cells following PIAS4 depletion indicate that inhibition of AMPKα1 SUMOylation could provide a specific way to inhibit cancers with hyperactive mTORC1 signalling.

## Methods

### Reagents

pFlag–PIAS1 (mouse), pFlag–PIASxα (rat), pFlag–PIASxβ (rat), pFlag–PIAS3 (mouse) and pFlag–PIAS4 (mouse) have been described^52^. pcDNA3-6His–SUMO1, pcDNA3-6His–SUMO2 and pcDNA3-6His–SUMO3 (human)^53^ was generously provided by Dr Ron Hay (University of Dundee, Dundee). pFlag–PIAS4-C335F was generated from pFlag–PIAS4 using Quickchange mutagenesis (Agilent) and primers are described in Supplementary Table 2. For expression of GST–AMPKα1 and GST–AMPKα2 in mammalian cells, AMPKα1 (human) and AMPKα2 (human) complementary DNAs in pENTR221 (Invitrogen) were transferred to pDEST27 (Invitrogen), to generate pDEST27-AMPKα1 and pDEST27-AMPKα2. For the screening of potential SUMOylation-deficient mutant of AMPKα1, lysine to arginine mutants of AMPKα1 (human) were generated from pDEST27-AMPKα1 by Quickchange mutagenesis and primers are listed in Supplementary Table 2. For lentiviral expression of AMPKα1, AMPKα1 cDNAs in pENTR221 were transferred to pLenti6/V5-DEST (Invitrogen), to generate pLenti6/V5-AMPKα1, and pLenti6/V5-AMPKα1-K118R was generated from pLenti6/V5-AMPKα1 by using Quickchange mutagenesis. For production of GST–TSC2 fragment (1,300–1,367) in bacteria, DNA fragments corresponding to amino acids 1,300–1,367 were amplified from pcDNA3-HA-TSC2 WT (rat) and pcDNA3-HA-TSC2 S1345A (rat) (Addgene), and transferred through pDONR221 to pDEST15 (Invitrogen), to generate pDEST15-TSC2 (1,300–1,367). For the production of GST–PIAS4 in bacteria, full-length PIAS4 cDNA was amplified from pFlag–PIAS4 (mouse) and transferred through pDONR221 to pDEST15, to generate pDEST15-PIAS4.

Antibodies against p-Raptor (Ser792, 2083, 1:1,000), Raptor (2280, 1:1,000), p-ACC (Ser79, 3661, 1:1,000), ACC (3662, 1:1,000), p-4EBP1 (Ser65, 9451, 1:1,000), 4EBP1 (9452, 1:1,000), p-S6K (Thr389, 9205, 1:1,000), S6K (9202, 1:1,000), p-S6 (Ser235/236, 2211, 1:10,000), S6 (2217, 1:1,000), p-AMPKα (Thr172, 2535, 1:1,000), AMPKα (2532, 1:1,000), AMPKβ1/2 (4150, 1:1,000), AMPKγ2 (2536, 1:1,000), TSC2 (4308, 1:1,000) and glyceraldehydes 3-phosphate dehydrogenase (GAPDH, 2118, 1:5,000) were purchased from Cell Signaling; p53 (sc-263, 1:1,500) were from Santa Cruz Biotech; AMPKα1 (ab32047; used in Fig. 1b for IP, in Figs 2a and 3a for WB, in Fig. 4a,b for IP and WB, and Fig. 4c,e, Supplementary Figs 1b,cd and 6a,c; WB dilution 1:1,000, IP dilution 1:50), AMPKα1 (ab110036, 1:1,000; used in Fig. 1b for WB), AMPKα2 (ab3760, WB dilution 1:1,000, IP dilution 1:100), GST (ab9085, 1:500; for immunofluorescence and PLA) and PIAS4 (ab58416, 1:1,000) from Abcam; also Flag M2 (F1804, Sigma, 1:2,000), SUMO1 (33–2,400, Invitrogen, 1:1,000), SUMO2/3 (M114-3, MBL, 1:500), GST (A00865, GenScript, for WB analysis, 1:5,000) and His (34660, Qiagen, 1:2,000) antibodies were used as indicated. PIAS2 rabbit antiserum (1:2,000) was described earlier^54^. Affinity-purified AMPKα1 antibodies^15^ (referred to as ‘H1'; used in Supplementary Fig. 1c for WB with a dilution of 1:1,000; used in Fig. 3a,c and Supplementary Fig. 1d for IP with a dilution of 1:35) for kinase assays were generously provided by Dr Grahame Hardie (University of Dundee).

AICAR was obtained from Toronto Research Chemicals (A611700); metformin hydrochloride (04635) and phenformin hydrochloride (P7045) were from Sigma; A769662 from Abcam (ab120335); rapamycin from Cell Signaling (9904); Slide-A-Lyzer Dialysis cassette (66380) and HisPur Ni-NTA magnetic beads from Pierce (88832); NEM (E3876), Trypan blue solution (T8154), L-arabinose (A3256) and L-Glutathione Reduced (G4251) from Sigma; Protein G Sepharose (17-0618) and Glutathione-Sepharose (17-0756) from GE Healthcare; SAMS peptide (12–355), P81 Phosphocellulose Squares (20–134) and recombinant human AMPK (α1, β1 and γ1) protein (14–840) from Millipore; ATP, [γ-^32^P] (3,000 Ci mmol^−1^, 10 mci ml^−1^) from PerkinElmer; BL21-A1 competent cells (C6070-03) from Invitrogen; Poly-Prep chromatography columns (731–1550) from Bio-rad; recombinant human p53 protein (SP-454-020) from R&D Systems; and SUMO3 conjugation kit (K-720) from Boston Biochem.

All siRNAs were purchased either as pools or individual siRNAs from Dharmacon and are listed in Supplementary Table 3.

Human pLKO.1-shPIAS4-5 (TRCN0000004115) and pLKO.1-shPIAS4-8 (TRCN0000004118) constructs were from the TRC collection^55^ and pLKO.1-shNT (SHC002) was from Sigma.

### Yeast two-hybrid screens

Yeast two-hybrid screens were performed using the Gateway ProQuest Two-Hybrid System (Invitrogen) essentially as described by the manufacturer. To generate bait plasmids, AMPKα1 (encoding amino acids 10–559) and AMPKα2 (full length) in pENTR221 (Invitrogen) were transferred to the pDEST32 (Invitrogen) and transformed into MAV203 *Saccharomyces cerevisiae* strain. The ProQuest pre-made human fetal brain cDNA library and a human liver cDNA library in EXP-AD502 (Invitrogen) were transformed into pDEST32-AMPKα1 or pDEST32-AMPKα2 yeast strains, to perform screenings. AMPKα1/brain (4.2 × 10^6^), AMPKα2/brain (2.9 × 10^6^), AMPKα1/liver (2.1 × 10^6^) and AMPKα2/liver (2.0 × 10^6^) colonies were screened and positive clones were subjected to sequencing.

### Cell culture and transfection

To generate immortalized MEFs, pregnant female mice were maintained in Laboratory Animal Center of the University of Helsinki. MEF isolation was approved by the National Animal Experiment Board in Finland. WT, *AMPKα1*^*+/−*^*;α2*^*lox/lox*^ and *AMPKα1*^−/−^*;α2*^*lox/lox*^ primary MEFs^56^ were isolated from E12.5 embryos and immortalized at passage 2 using the carboxy terminus of p53 in pBabe-Hygro^57^ and hygromycin (200 μg ml^−1^) selection for 7 days. Immortalized *AMPKα1*^*+/−*^*;α2*^*lox/lox*^ and *AMPKα*^−/−^*;α2*^*lox/lox*^ MEFs were then transduced with adenoviral vectors expressing green fluorescent protein or Cre recombinase^58^, to obtain immortalized *AMPKα1*^*+/−*^*;α2*^*+/+*^, *AMPKα1*^*+/−*^*;α2*^−/−^, *AMPKα1*^−/−^*;α2*^*+/+*^ or *AMPKα1*^−/−^*;α2*^−/−^ MEFs, respectively. NIH3T3, HEK293, HEK293FT and MDA-MB-231 cells were obtained from ATCC and maintained in DMEM medium supplemented with 10% fetal bovine serum (Gibco), 1% L-glutamine and antibiotics (penicillin and streptomycin). All the cells have been tested to be free of mycoplasma contamination. siRNAs were transfected twice at a final concentration of 40 nM by using Lipofectamine 2000 (Invitrogen) on day 0 and day 1 according to suggested protocol; plasmid transfections were performed on day 1 or when combined with siRNA transfections on day 2 using Lipofectamine 2000. Cells were collected on day 2 (only plasmid) or day 3 for analysis.

### Real-time quantitative PCR

Total RNAs from cell lysates were isolated using the RNeasy isolation kit (Qiagen) according to manufacturer's instructions and reverse transcribed using Taqman MULV reverse transcriptase and random hexamers (Applied Biosystems). Real-time quantitative PCR was performed with StepOnePlus RT-PCR System (Applied Biosystems) using SYBR-green RT-PCR reagent (Applied Biosystems) and primers listed in Supplementary Table 4. Relative mRNA levels were calculated relative to mouse or human GAPDH.

### IP and purification of GST-tagged proteins

For IP, cells were lysed in IP lysis buffer (50 mM Tris pH 7.4, 150 mM NaCl, 1 mM EDTA, 1 mM EGTA, 50 mM NaF, 1% Triton X-100, 1 mM dithiothreitol (DTT), 10 μg ml^−1^ Aprotinin, 0.1 mM phenylmethyl sulfonyl fluoride (PMSF), 10 μg ml^−1^ Leupeptin, 10 mM β-glycerophosphate and protease inhibitor cocktail (Roche)) and lysates were cleared by centrifugation (10 min at 16,000*g*, +4 °C) followed by IP with antibody against AMPKα1 or AMPKα2 and protein G-Sepharose, and five washes with lysis buffer, and subsequently subjected for kinase assays or WB analysis.

For purification of GST-tagged proteins from transfected HEK293, cells were lysed in ELB buffer (50 mM HEPES pH 7.4, 150 mM NaCl, 5 mM EDTA, 0.1% NP-40, 1 mM DTT, 2.5 μg ml^−1^ aprotinin, 0.5 mM PMSF, 10 mM β-glycerophosphate and 1 μg ml^−1^ leupeptin). Cleared (10 min at 16,000*g*, +4 °C) cell lysates were incubated with Glutathione-Sepharose overnight at +4 °C, then by washing the beads five times with ELB buffer followed by kinase assay or WB analysis. The sample size for each condition is 1 (*n*=1) within one experiment.

### Nuclear/cytoplasmic fractionation

Nuclear/cytoplasmic fractionation was performed using NE-PER Nuclear and Cytoplasmic Extraction Reagents (ThermoFisher) and nuclear fractions were extracted using ELB buffer (50 mM HEPES pH 7.4, 150 mM NaCl, 5 mM EDTA, 0.1% NP-40, 1 mM DTT, 2.5 μg ml^−1^ aprotinin, 0.5 mM PMSF, 10 mM β-glycerophosphate and 1 μg ml^−1^ leupeptin) instead of NER buffer provided by the kit. The fractions were analysed following purification with GST pulldown or analysed directly by WB. The sample size for each condition is 1 (*n*=1) within one experiment.

### WB analysis

For total protein analysis, cells were lysed using SDS boiling buffer (2.5% SDS, 250 mM Tris pH 6.8, including 50 mM NaF, 10 mM β-glycerophosphate, 0.5 mM DTT, 0.5 mM PMSF) and lysates were boiled at +98 °C and needled (25-gauge) ten times. Lysates were then cleared by centrifugation and protein concentration was measured by using Bio-Rad DC protein assay (Bio-Rad). Twenty micrograms of protein lysates were used for WB. For WB analysis, 8–12% SDS–PAGE gels were transferred to nitrocellulose membrane and blotted according to the antibody manufacturer's instructions. Immunoblots were developed using SuperSignal West Femto Maximum Sensitivity Substrate (34096, Thermo). Blots were cropped to include at least one marker position. Uncropped blots for all main figures were included in Supplementary Figs 9–14. The sample size for each condition is 1 (*n*=1) within one experiment.

### Immunofluorescence analysis and quantification

Cells on coverslips were fixed with 4% paraformaldehyde (PFA) for 15 min and then permeabilized using 0.1% Triton X-100 for 5 min. Cells were then blocked with 5% goat serum in PBS for 30 min, incubated with anti-GST antibody (1:500) for 30 min, washed three times with PBS, incubated with Alexa594 secondary antibody for 30 min, washed three times with PBS, labelled with 4,6-diamidino-2-phenylindole (DAPI) and mounted with Immuno-mount (Thermo Scientific).

Stained coverslips were analysed and imaged using Zeiss AxioImager.M2 fluorescent microscope. Transfected cells were identified by thresholding Alexa594 image using ImageJ 1.49a. Total GST–AMPKα1 levels were measured as the integrated density within the identified transfected cells. Nuclei were identified by DAPI stain. Nuclear levels of GST–AMPKα1 were measured as the integrated density within the nuclear region and expressed as a percentage of total GST–AMPKα1 for each identified transfected cells. The mean nuclear levels of GST–AMPKα1-WT and GST–AMPKα1-K118R cells from three equally weighed independent experiments were compared with Student's *t*-test. The sample size for each condition is 1 (*n*=1) within one experiment.

### PLA analysis and quantification

PLAs were performed using Duolink In Situ Red Starter Kit Mouse/Rabbit (DUO92101, Sigma) according to the manufacturer's protocol. Briefly, cells were fixed with 4% PFA for 15 min and then permeabilized using 0.1% Triton X-100 for 5 min. After blocking with 5% goat serum in PBS for 30 min, cells were labelled with anti-GST (rabbit, 1:500) and anti-SUMO2/3 (mouse, 1:500) for 30 min. After washing with PBS for three times, cells were labelled with PLA probe plus and minus diluted in PBS for 1 h in a pre-heated humidity chamber. Ligation and amplification were performed as detailed by the manufacturer. Slides were mounted with Duolink Mounting medium with DAPI and were imaged using Zeiss AxioPlan2 with × 10 objectives.

Forty images per coverslip of two independent experiments were analysed using ImageJ 1.49a. Cell nuclei were identified by DAPI stain (area size between 400–3,000 pixels). Regions of interest (ROIs) for PLA signal measurement of individual cells were created by dilating cell nuclei with 20 iterations followed by the watershed algorithm. GST transfected control was used to correct for background PLA signal by using it as a reference to set a threshold for PLA signal (imaged under Alexa 594 channel), where minimal signal is obtained in GST-transfected control. The same threshold was then applied to other samples. PLA signal was measured as arbitrary unit of integrated density within the ROIs. Integrated density for individual cells within GST-transfected control were measured and the maximum value (GSTmax) was used as cutoff, whereby cells with integrated density greater than GSTmax are identified as positive for PLA signal. Fisher's exact test was used for statistical analysis of percentage of positive cells.

PLA signal is also measured within the nuclear region of each cells and percentage of nuclear PLA signal is expressed as integrated density within the nuclear region divided by integrated density within the ROIs multiplied by 100. Student's *t*-test was used for statistical analysis.

Transfection efficiency was determined from coverslips (from the same transfection of coverslips used for PLA) stained for anti-GST antibody, five images per coverslips from two biological experiments. Cells that were clearly non-transfected were used as background reference for thresholding GST signal to a black and white binary image, of which pixels with intensity value above the threshold value is white and those below is black. A single threshold value is used for all samples. Each cell, represented by ROIs created as described above, is classified as transfected cells when it has more than 500 pixels of white. The appropriateness of such analysis was confirmed manually by the eye. Fisher's exact test was used for statistical analysis of transfection efficiency. The sample size for each condition is 1 (*n*=1) within one experiment.

### Protein purifications from bacteria

pDEST15-TSC2 (1,300–1,367) and pDEST15-PIAS4 were transformed into BL21-A1 cells. Expression of GST–TSC2 (1,300–1,367) was induced with L-arabinose at a final concentration of 0.2% for 4 h at +37 °C and expression of GST–PIAS4 was induced with L-arabinose at a final concentration of 0.2% for 16 h at +16 °C. Cell pellets were resuspended in cold lysis buffer (PBS, 1% Triton X-100, 1 mg ml^−1^ lysozyme, 25 mM PMSF, 10 μg ml^−1^ Aprotinin, 10 μg ml^−1^ Leupeptin). Lysates were sonicated and centrifuged (30 min, at 16,000, +4 °C), and supernatants were rotated with glutathione-sepharose beads in Poly-Prep chromatography columns at +4 °C for 2 h. Packed beads were washed three times with PBS followed by elution in 10 mM L-Glutathione Reduced, 50 mM Tris pH 8.0 (for GST–TSC2 (1,300–1,367)) or 10 mM L-Glutathione Reduced, 50 mM Tris pH 8.0, supplemented with 5 mM DTT (for GST–PIAS4). Eluted proteins (GST–TSC2 (1,300–1,367)) were dialysed against Tris-buffered saline using Slide-A-Lyzer Dialysis cassette (10-kDa cutoff) at +4 °C overnight and stored at −70 °C.

### Kinase assays

Cells to be analysed were replenished with fresh DMEM medium with or without 2 mM AICAR for 2 h before lysing in ice-cold IP lysis buffer. Twenty-five micrograms of cleared lysates were incubated with 1 μl of affinity-purified AMPKα1 antibody (H1) at +4 °C with rotation overnight followed by addition of 20 μl of a 1:1 slurry of Protein G Sepharose prewashed with IP lysis buffer. Beads were washed three times with cold IP lysis buffer and two times with cold HEPES-Brij buffer (50 mM HEPES pH 7.4, 1 mM DTT, 0.02% Brij-35) followed by a kinase assay in 50 mM HEPES pH 7.4, 1 mM DTT, 0.02% Brij-35, 5 mM MgCl_2_, 5 μCi [γ-32P] ATP 3,000 Ci mmol^−1^ and 2 μg GST–TSC2 (1,300–1,367) or 2.5 μg SAMS peptide in a final reaction volume of 25 μl at +30 °C for 30 min. For the kinase assay using GST–TSC2 fragment as substrates, reactions were terminated with 25 μl 2 × Laemmli sample buffer (65.8 mM Tris-HCl pH 6.8, 2.1% SDS, 26.3% (w/v) glycerol, 0.01% bromophenol blue), boiled at +98 °C for 5 min and subjected to SDS–PAGE and autoradiography. For the kinase assay using SAMS peptide as substrates, reactions were terminated by spotting 10 μl reaction mixture to the centre of P81 phosphocellulose square. P81 squares were then washed two times with 1% phosphoric acid buffer, one time with H_2_O and one time with acetone. Dried P81 squares were transferred to scintillation vial containing 2 ml scintillation cocktail and radioactivity were measured with scintillation counter from three independent experiments. The sample size for each condition is 1 (*n*=1) within one experiment. Student's *t*-test was used for statistical analysis.

### SUMOylation assay

Purification of 6His-tagged SUMO under denaturing condition was performed according to the protocol described previously^53^ with slight modification. HEK293 cells (10 cm plate) were transfected with cDNAs and 24 h later cells were washed once with 10 ml ice-cold PBS containing 20 mM NEM, and washed cells were scraped off from the plate with another 5 ml ice-cold PBS containing 20 mM NEM. For the Input sample preparation, 500 μl of cell suspension were transferred to 1.5 ml tube and spinned at 1,000*g* for 2 min. Cell pellet was re-suspended with 100 μl 1 × Laemmli sample buffer, needled and boiled at +98 °C for 5 min before loading to SDS–PAGE. To prepare the sample for nickel affinity purification, rest of the cell suspension were centrifuged at 1,000*g* and re-suspended with 5 ml freshly prepared Lysis buffer (0.1 M Na_2_HPO_4_/NaH_2_PO_4_ pH 8.0, 6 M Guanidinium-HCl, 10 mM Tris pH 8.0, 0.05% Tween). Samples were sonicated for 30 s on low power on ice and cleared by centrifugation at 16,000*g*, +4 °C for 10 min. Supernatants were then diluted with equal volume of Equilibration buffer (0.1 M Na_2_HPO_4_/NaH_2_PO_4_ pH 8.0, 6 M Guanidinium-HCl, 10 mM Tris pH 8.0, 0.05% Tween, 30 mM Imidazole) and incubated overnight with rotation at +4 °C with 100 μl HisPur Ni-NTA magnetic beads prewashed with Equilibration buffer. Beads were then washed and collected on a magnetic stand three times with Wash buffer 1 (0.1 M Na_2_HPO_4_/NaH_2_PO_4_ pH 8.0, 6 M Guanidinium-HCl, 10 mM Tris pH 8.0, 0.05% Tween, 30 mM Imidazole) and three times with Wash buffer 2 (0.1 M Na_2_HPO_4_/NaH_2_PO_4_ pH 8.0, 10 mM Tris pH 8.0, 0.05% Tween, 30 mM Imidazole, 8 M urea). His-tagged proteins were finally eluted with 100 μl Elution buffer (200 mM imidazole, 5% SDS, 150 mM Tris-Cl pH 6.7, 30% glycerol, 720 mM β-ME, 0.0025% bromophenol blue) for 20 min at room temperature and 1 min at +98 °C, and 20 μl protein elutions were resolved by 8% SDS–PAGE gel.

*In vitro* SUMOylation was performed according to the manual provided by SUMO3 conjugation kit (K-720, Boston Biochem) with a slight modification. Briefly, 9 μl of glutathione elution buffer (10 mM L-Glutathione Reduced, 50 mM Tris pH 8.0, supplemented with 5 mM DTT) with or without GST–PIAS4, 2 μl 10 × Reaction buffer, 1 μl p53 protein (285 ng) or 0.5 μl AMPK (α1, β1 and γ1) protein (500 ng), 2 μl SUMO E1 enzyme, 2 μl SUMO E2 enzyme, 2 μl SUMO3, 2 μl 10 × Mg^2+^-ATP Solution were mixed in a 20-μl volume and the reactions were carried out in +37 °C for 1 h. The reactions were terminated with addition of 5 × SDS–PAGE sample buffer and 1 μl 1 M DTT, and boiling at 98 °C for 5 min. Five-microlitre reactions were resolved by 10% SDS–PAGE gel and blotted with either p53 or AMPKα1 antibody. The sample size for each condition is 1 (*n*=1) within one experiment.

### Lentivirus production and transduction

For the production of lentivirus, lentiviral constructs (see Reagents) were co-transfected with VSV-G and Delta 8.9 packaging plasmids (Invitrogen) into HEK293FT cells using Lipofectamine 2000, followed by collection of supernatant 48 h later and filtering through a 0.45-μm filter (Millipore). For the lentiviral transduction, 1 × 10^5^ immortalized *AMPKα1*^−/−^*;α2*^−/−^ MEFs or 1 × 10^6^ MDA-MB-231 cells were seeded on six-well plates and transduced the following day with lentiviral supernatants in the presence of 8 μg ml^−1^ polybrene overnight. Transduced *AMPKα1*^−/−^*;α2*^−/−^ MEFs were selected with (10 μg ml^−1^) blasticidin (Invitrogen) and MDA-MB-231 cells were selected with (2.5 μg ml^−1^) puromycin (Gibco) for 1 week.

### Growth inhibition assay in MDA-MB-231 cells

Selected pools of lentiviral-transduced MDA-MB-231 cells were seeded as triplicates (*n*=3) (1.5 × 10^5^ cells per well) into 24-well plates. The following day (day 1) cells were treated with vehicle control (dimethyl dulfoxide), A769662 (150 μM), rapamycin (10 μM) or A769662 (150 μM)+rapamycin (10 μM). Cell proliferation was assessed by comparing number of viable cells on day 2, 3 and 4 with those of day 1 using Trypan blue exclusion and a TC20 cell counter (Bio-Rad) from three independent experiments. Student's *t*-test was used for statistical analysis. The sample size for each condition is 3 (*n*=3) within one experiment.

### Quantification of blots and statistics

All experiments have been done at least twice and representative results are shown. Densitometric analysis of WB and 32P radioautography results was done using Image J 1.49a (NIH). Intensity of each band was measured and subtracted with background intensity from adjacent area. Values in the results section were presented as relative intensity versus control. Statistical analyses were performed either by using the analysis of variance followed by Fisher's least significant difference test (GraphPad Prism version 6.0c for Mac OS X) (for Fig. 1d) or by two-sided Student's *t*-test (for all the other quantifications). Data were presented as mean+s.e.m. Statistical significance was set at *P*-values of NS>0.05, *<0.05, **<0.01 and ***<0.001.

## Additional information

**How to cite this article:** Yan, Y. *et al*. SUMOylation of AMPKα1 by PIAS4 specifically regulates mTORC1 signalling. *Nat. Commun.* 6:8979 doi: 10.1038/ncomms9979 (2015).

## Supplementary Material

Supplementary Information Supplementary Figures 1-14 and Supplementary Tables 1-4
