# Supplementary material for: SUMOylation of AMPKα1 by PIAS4 specifically regulates mTORC1 signalling
Source: Nat Commun. 2015 Nov 30;6:8979. doi: 10.1038/ncomms9979 (PMC4674823; doi:10.1038/ncomms9979)
Supplement: Supplementary Information — Supplementary Figures 1-14 and Supplementary Tables 1-4 [file ncomms9979-s1.pdf]

Supplementary Figure-1 (Makela)

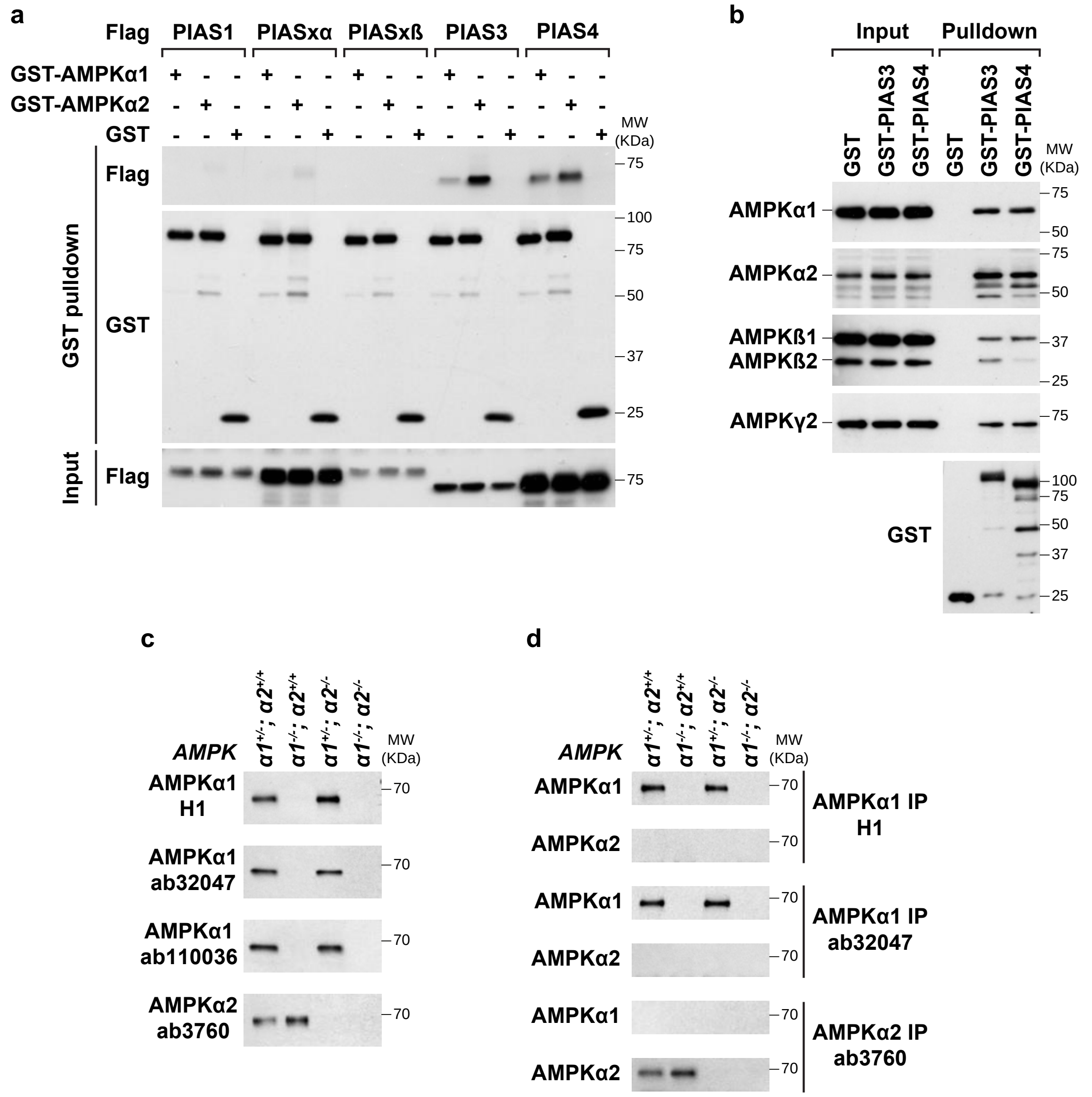

Supplementary Figure 1. Association of PIAS3 and PIAS4 with endogenous AMPKα, AMPKß, and AMPKγ subunits. (**a**) (Upper panels) Lysates of HEK293 cells cotransfected with plasmids encoding one of the Flag-tagged PIAS family proteins (PIAS1, PIASxα, PIASxß, PIAS3, or PIAS4) together with plasmids encoding either GST-AMPKα1 or GST-AMPKα2 as indicated were analyzed 24h later following purification with glutathione-sepharose (GST pulldown) by Western blotting analysis with Flag or GST antibodies. (Lower panel) Western blotting analysis of total lysates (Input) with the indicated antibodies. (**b**) Lysates of HEK293 cells transfected with plasmids encoding GST, GST-PIAS3, or GST-PIAS4 were analyzed 24h later directly (Input) or following purification with glutathione-

sepharose (Pulldown) by Western blotting analysis with antibodies indicated on the left. (**c**) Cell lysates from immortalized MEFs with variable *AMPKα* genotypes indicated on the top were analyzed by Western blotting using antibodies indicated on the left. (**d**) Immortalized MEFs with indicated *AMPKα* genotypes on the top were subjected for immunoprecipitation (IP) with indicated AMPKα1 or AMPKα2 antibody shown on the right and subsequently analyzed by Western blotting using AMPKα1 (ab110036, Abcam) or AMPKα2 (ab3760, Abcam) antibodies indicated on the left.

Supplementary Figure-2 (Makela)

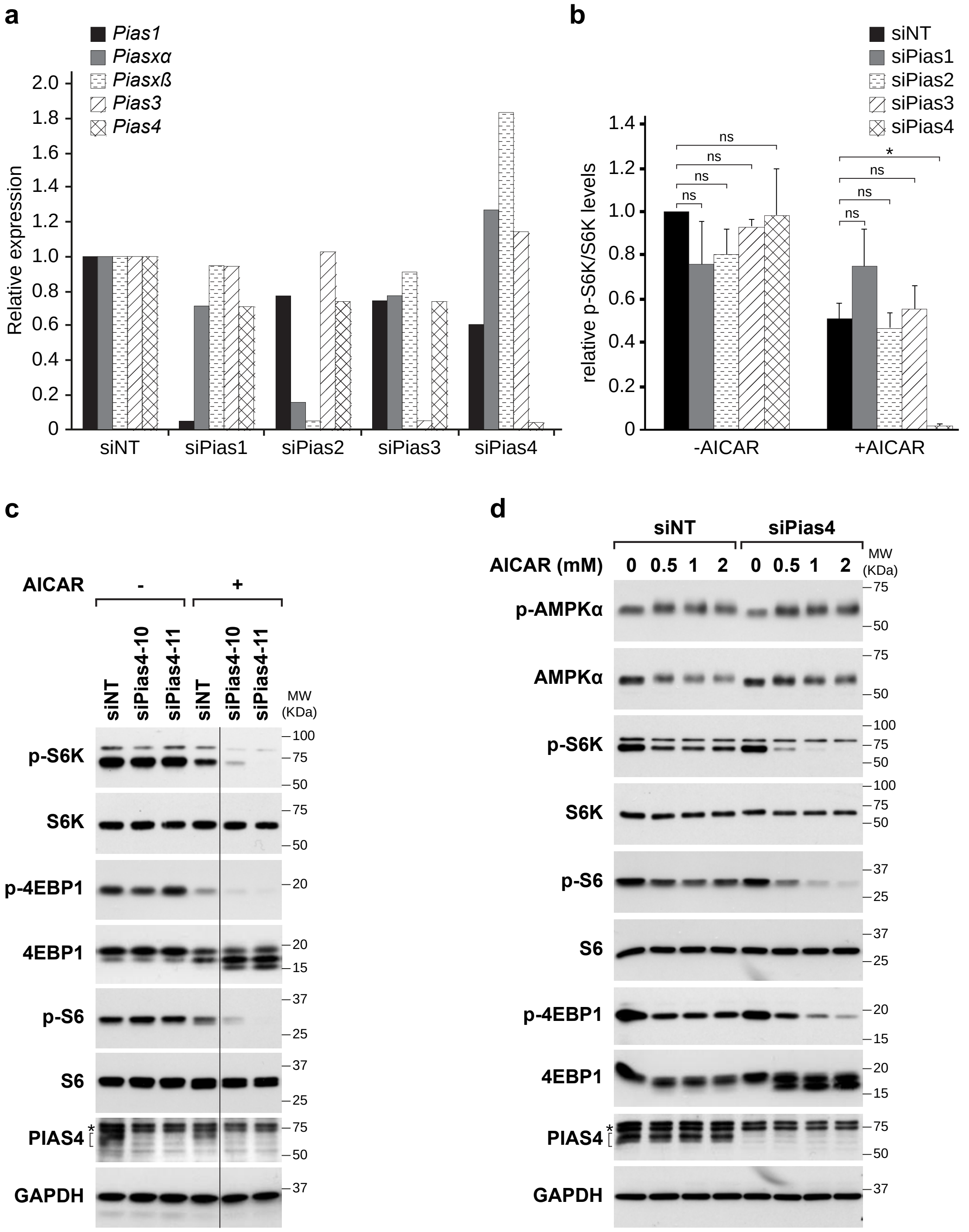

Supplementary Figure 2. PIAS4 attenuates AMPK inhibition of mTORC1 signaling. (**a**) Relative mRNA levels of *Pias1*, *Piasxα*, *Piasxß*, *Pias3* and *Pias4* were analyzed by qRT-PCR from immortalized MEFs transfected with nontargeting siRNA pool (siNT) or siRNA pools against the indicated mouse Pias genes. (**b**) Relative levels of p-S6K/S6K of immortalized MEFs transfected with indicated siRNAs followed by treatment with vehicle (-AICAR) or 2 mM AICAR (+AICAR). Data are shown as means+SEM (n=2) relative to the –AICAR condition in siNT transfected

cells (with representative images in Fig. 1a). Significances of differences by Student's t-test are indicated (*P<0.05, ns>0.05). (**c**) Immortalized MEFs were transfected with siNT pool or with two independent siRNAs against mouse Pias4 (siPias4-10 and siPias4-11). At 72h post-transfection, cells were treated with vehicle or 2 mM AICAR for 2h and cell lysates were analyzed by Western blotting using the indicated antibodies. For PIAS4 blot, asterisk denotes unspecific signals and bracket denotes specific signals. (**d**) Immortalized MEFs were transfected with siRNA pools indicated on the top, and 72 hours later treated with the indicated concentrations of AICAR for 2 h following analysis as in (**c**). For PIAS4 blot, the asterisk denotes unspecific signals and the bracket denotes specific signals.

Supplementary Figure-3 (Makela)

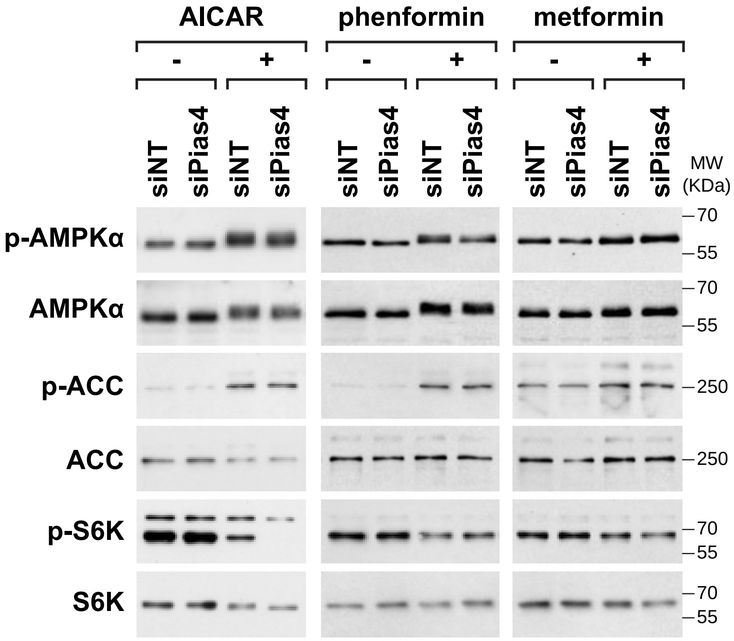

Supplementary Figure 3. PIAS4 depletion does not affect mTORC1 signaling upon phenformin or metformin treatment. Immortalized MEFs transfected with siRNA pools indicated on the top were treated with 2 mM AICAR for 2 hours or 5 mM phenformin for 1 hour or 1 mM metformin for 24 hours, and cell lysates were analyzed by Western blotting using the indicated antibodies.

Supplementary Figure-4 (Makela)

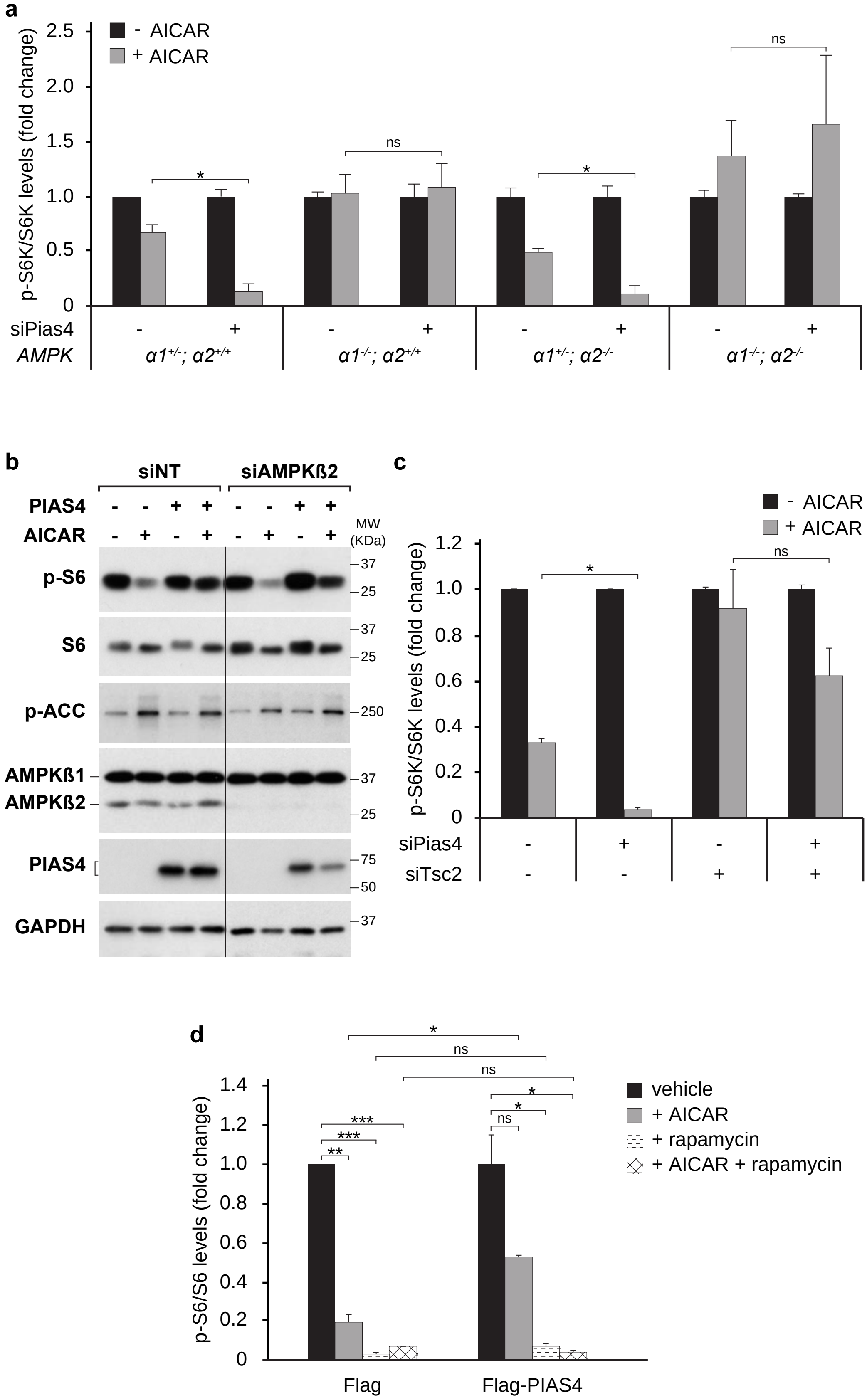

Supplementary Figure 4. PIAS4 modulates mTORC1 signaling via AMPKα1 and TSC2. (**a**) Relative levels of p-S6K/S6K in immortalized MEFs with variable *AMPKα* genotypes transfected with siNT or siRNA pool against Pias4 (siPias4) followed by treatment with vehicle (-AICAR) or 2 mM AICAR (+AICAR). Data are shown as means+SEM (n=2) relative to the –AICAR conditions (with representative images in Fig. 2a). Significances of differences by Student's t-test are indicated (*P<0.05, ns>0.05). (**b**) NIH3T3 cells were initially transfected with siNT pool or siRNA pool against mouse Prkab2 (AMPKß2) (siAMPKß2) and 24h later with vector (Flag) or Flag-PIAS4 encoding plasmids. 24h later cells were treated with vehicle (-) or 2 mM AICAR (+) for 2h, and lysed for SDS-PAGE and Western blotting analysis with the indicated antibodies. (**c**) Relative levels of p-S6K/S6K in immortalized MEFs transfected with siPias4 pool or siRNA pool against mouse Tsc2 (siTsc2) or both as indicated followed by treatment with vehicle (-AICAR) or 2 mM AICAR (+AICAR). Data are shown as means+SEM (n=2) relative to the –AICAR conditions (with representative images in Fig. 2b). Significances of differences by Student's t-test are indicated (*P<0.05, ns>0.05). (**d**) Relative levels of p-S6/S6 in NIH3T3 cells transfected with vector control (Flag) or Flag-PIAS4 plasmids followed by treatment with vehicle, 2 mM AICAR (+AICAR), 50 nM rapamycin (+rapamycin) or both (+AICAR+rapamycin). Data are shown as means+SEM (n=2) relative to the vehicle control conditions (with representative images in Fig. 2c). Significances of differences by Student's t-test are indicated (***<0.001, **<0.01, *P<0.05, ns>0.05).

Supplementary Figure-5 (Makela)

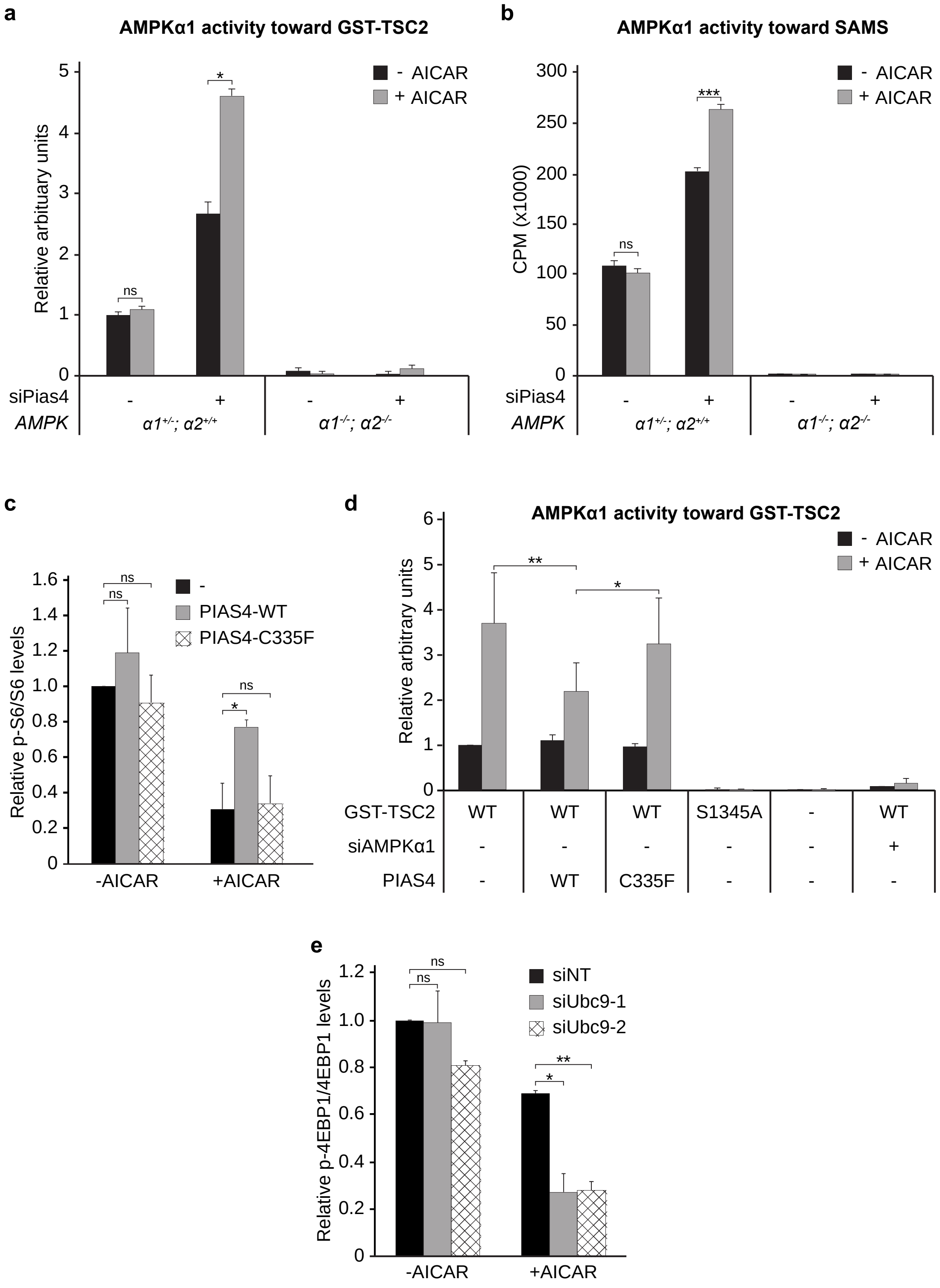

Supplementary Figure 5. PIAS4 inhibits AMPKα1 kinase activity in a SUMO E3 ligase dependent manner. (**a**) Relative arbitrary units (means + SEM, n=3) of 32P autoradiography intensity (See Methods) of AMPKα1 kinase assay using GST-TSC2 recombinant proteins as substrates (with representative images in Fig. 3a). Significances of differences by Student's t-test are indicated (*P<0.05, NS>0.05). (**b**) Immortalized *AMPKα1*$^{+/-}$*;α2*$^{+/+}$ *or AMPKα1*$^{-/-}$*;α2*$^{-/-}$ MEFs indicated at the bottom of each chart were transfected with siNT pool or siPias4 pool and 72h later cells were treated with vehicle (-) or 2 mM AICAR for 2h. 25μg of cell lysates from each treatment were used for AMPKα1 immunoprecipitation and immunoprecipitates were used to phosphorylate 2.5μg SAMS peptide *in vitro* in the presence of [γ-^{32}P] ATP followed by scintillation counting. Data are means+SEM (n=3). Significances of differences by Student's t-test are indicated (***P<0.001, NS>0.05). (**c**) Relative levels of p-S6/S6 in NIH3T3 cells transfected with vector control (-), Flag-PIAS4-WT (WT) or Flag-PIAS4-C335F (C335F) plasmids followed by treatment with vehicle (-AICAR) or 2 mM AICAR (+AICAR). Data are shown as means+SEM (n=2) relative to the -AICAR condition in Flag transfected cells (with representative images in Fig. 3b). Significances of differences by Student's t-test are indicated (*P<0.05, ns>0.05). (**d**) Relative arbitrary units (mean+SEM, n=3) of 32P autoradiography intensity (See Methods) of AMPKα1 kinase activity using GST-TSC2 recombinant proteins as substrates (with representative images in Fig. 3c). Significances of differences by Student's t-test are indicated (**P<0.01, *P<0.05). (**e**) Relative levels of p-4EBP1/4EBP1 in immortalized MEFs transfected with siNT pool or two independent siRNAs against mouse Ubc9 (siUbc9-1 and siUbc9-2) followed by treatment with vehicle (-AICAR) or 2 mM AICAR (+AICAR). Data are shown as means+SEM (n=2) relative to the -AICAR condition in siNT transfected cells (with representative images in Fig. 3d). Significances of differences by Student's t-test are indicated (**P<0.01, *P<0.05, ns>0.05).

Supplementary Figure-6 (Makela)

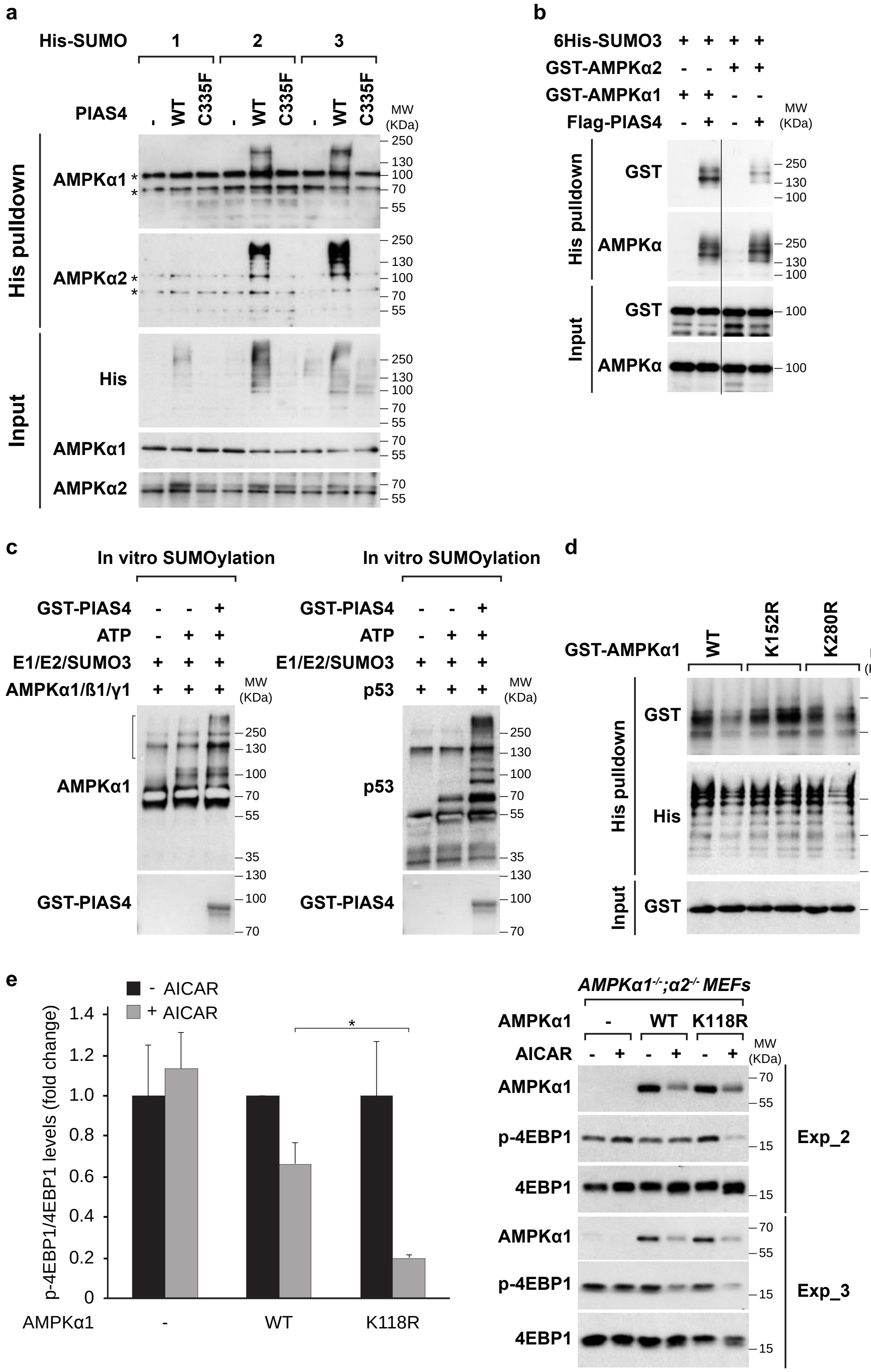

Supplementary Figure 6. SUMOylation of AMPKα1 and AMPKα2 by SUMO2 and SUMO3. (**a**) **(**Upper panels) Western blotting analysis using anti-AMPKα1 or anti-AMPKα2 antibodies from His-pulldown prepared from denaturing lysates of HEK293 cells co-transfected with plasmids encoding 6His-SUMO1 or SUMO2 or SUMO3 and either Flag (-) or Flag-PIAS4-WT (WT) or Flag-PIAS4-C335F (C335F). (Lower panels) Western blotting analysis of total lysates (Input) with the indicated antibodies. The asterisks denote unspecific signals. (**b**) **(**Upper panels) Western blotting analysis using anti-GST or anti-AMPKα antibodies from His-pulldown prepared from denaturing lysates of HEK293 cells co-transfected with plasmids encoding 6His-SUMO3 and either Flag (-) or Flag-PIAS4 (+) and either GST-AMPKα1 or GST-AMPKα2. (Lower panels) Western blotting analysis of total lysates (Input) with the indicated antibodies. (**c**) (Left panels) Western blotting analysis using anti-AMPKα1 antibody from *in vitro* SUMOylation assay assembled using recombinant AMPK (α1, ß1, γ1) protein and recombinant SUMO E1, E2, SUMO3 in the absence or presence of GST-PIAS4 or ATP. GST-PIAS4 proteins were visualized with Coomassie Blue stain in the lower panel. Increase of AMPKα1 SUMOylation by GST-PIAS4 was noted at high molecular weight denoted by bracket. (Right panels) Western blotting analysis using anti-p53 antibody from *in vitro* SUMOylation assay assembled as described above using recombinant p53 as substrates in the absence or presence of GST-PIAS4 or ATP. GST-PIAS4 proteins were visualized with Coomassie Blue stain in the lower panel. (**d**) **(**Upper panels) Western blotting analysis using anti-GST or anti-His antibodies from His-pulldown prepared from denaturing lysates of HEK293 cells co-transfected with plasmids encoding 6His-SUMO3 and Flag-PIAS4 and either WT or K152R or K280R form of GST-AMPKα1. (Lower panel) Western blotting analysis of total lysates (Input) with the anti-GST antibodies. (**e**) (Left panels) Relative levels of p-4EBP1/4EBP1 of control vector (-), AMPKα1-WT (WT) or AMPKα1-K118R (K118R) reconstituted *AMPKα1*$^{-/-}$*;α2*$^{-/-}$ MEFs treated with vehicle (-AICAR) or 2 mM AICAR (+AICAR). Data are shown as means+SEM (n=3) relative to the –AICAR conditions (with representative images in Fig. 4e). Significances of differences by Student's t-test are indicated (*P<0.05). (Right panels) Western blotting analysis of cell lysates from two additional independent experiments (denoted by Exp_2 and Exp_3) using AMPKα1, p-4EBP1 and 4EBP1 antibodies.

Supplementary Figure-7 (Makela)

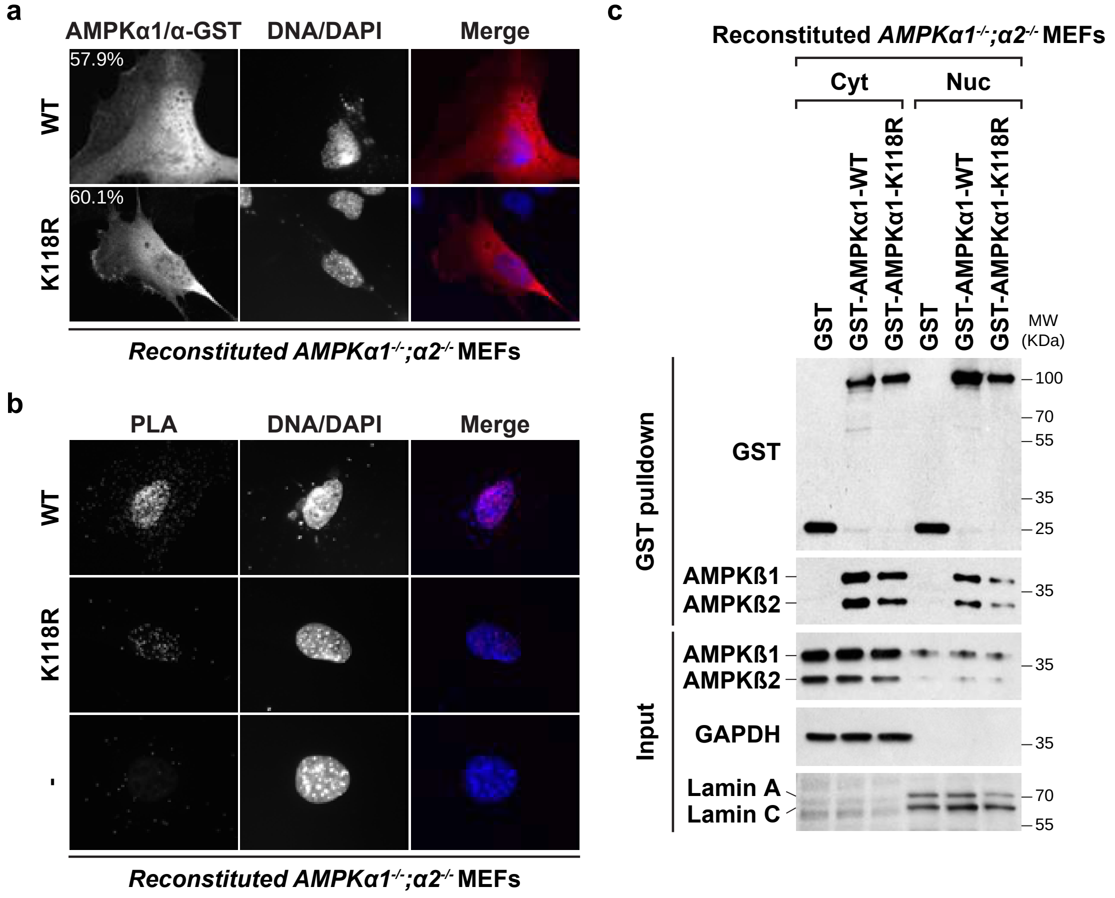

Supplementary Figure 7. Effects of SUMOylation on AMPKα1 subcellular localization and localization of SUMOylated AMPKα1 detected by proximity ligation assay (PLA). (**a**) Anti-GST immunofluorescence staining of immortalized *AMPKα1*$^{-/-}$*;α2*$^{-/-}$ MEFs transfected with GST-AMPKα1-WT or GST-AMPKα1-K118R followed by 2h treatment with 2 mM AICAR. DNA was stained with DAPI. The percentages on the upper-left corner show the quantification of cytoplasmic GST-AMPKα1 expressed as a percentage of total GST-AMPKα1 for each identified transfected cells. The mean of cytoplasmic GST-AMPKα1 for wildtype (WT) (696 cells) and K118R (842 cells) from 3 equally weighed independent experiments were compared with Student's t-test (*P<0.05). (**b**) Immortalized *AMPKα1*$^{-/-}$*;α2*$^{-/-}$ MEFs transfected with GST (-), GST-AMPKα1-WT (WT) or GST-AMPKα1-K118R (K118R) indicated on the left followed by treatment with 2 mM AICAR for 2 h were subjected for proximity ligase assay (PLA) using rabbit anti-GST and mouse anti-SUMO2/3 specific antibodies. DNA was stained with DAPI. PLA signal was not detected from control (-) MEFs, whereas a strong and predominantly nuclear PLA signal (83.4% nuclear PLA signal vs 16.6% cytoplasmic PLA signal) was detected in AMPKα1-WT cells. Quantification of PLA signal (See Methods) in AMPKα1-K118R showed less PLA positive cells and weaker PLA signal per positive cells, resulting in a decrease in total PLA signal in AMPKα1-K118R to 3% of that in AMPKα1-WT (PLA positive cells: 1.4% in AMPKα1-K118R vs 16.3% in AMPKα1-WT, p<0.0001, Student's t-test; transfection efficiency: 21% GST positive cells in both AMPKα1-WT and AMPKα1-K118R; mean integrated density of PLA per positive cell: 16445 in AMPKα1-K118R vs 54273 in AMPKα1-WT, AMPKα1-K118R is 30% of AMPKα1-WT, p<0.01, Student's t-test). (**c**) Immortalized *AMPKα1*$^{-/-}$*;α2*$^{-/-}$ MEFs transfected as in (**b**) were treated with 2 mM AICAR for 2 h followed by cytoplasmic (Cyt)-nuclear (Nuc) fractionation. The fractions were analyzed following purification with glutathione-sepharose (GST pulldown) or analyzed directly (Input) by Western blotting with antibodies indicated on the left.

Supplementary Figure-8 (Makela)

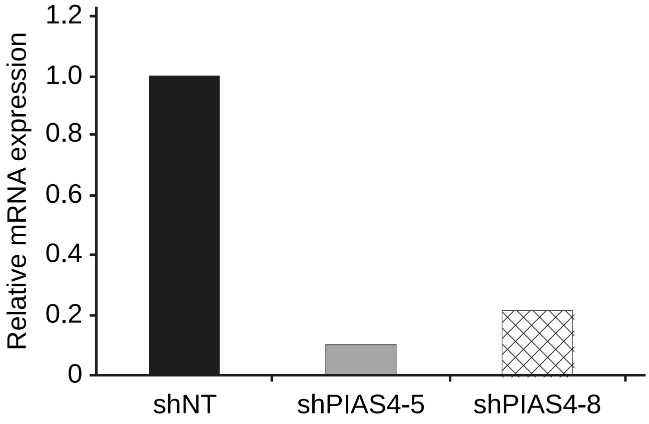

Supplementary Figure 8. Depletion of PIAS4 in MDA-MB-231 breast cancer cells. The level of *PIAS4* mRNA relative to *GAPDH* was assessed by RT-qPCR from the indicated lentivirus transduced MDA-MB-231 cells following selection with puromycin (2.5 μg/ml) for 1 week.

Supplementary Figure-9 (Makela)

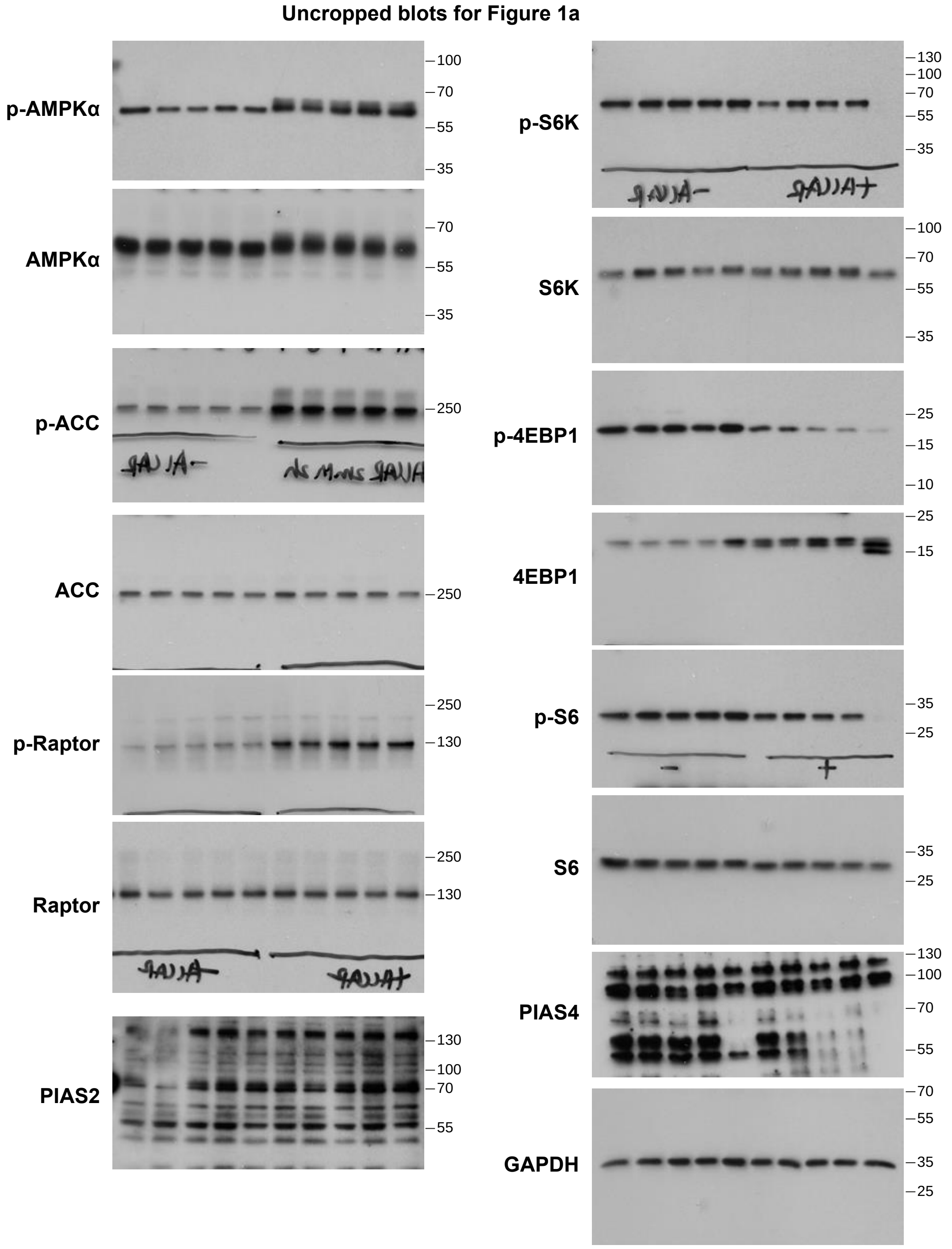

Supplementary Figure 9. Uncropped blots for Figure 1a

Supplementary Figure-10 (Makela)

Uncropped blots for Figure 1b

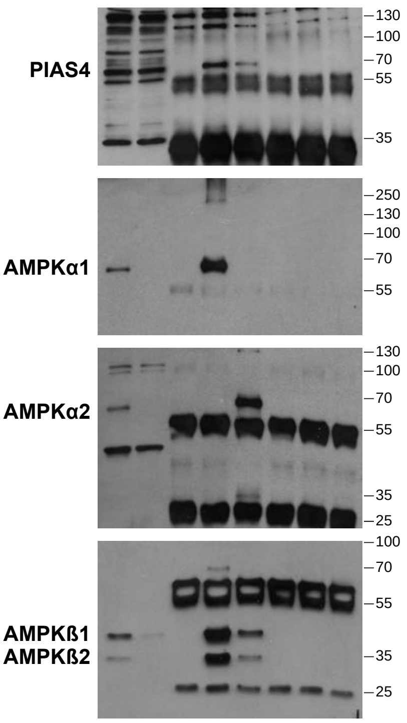

Uncropped blots for Figure 1c

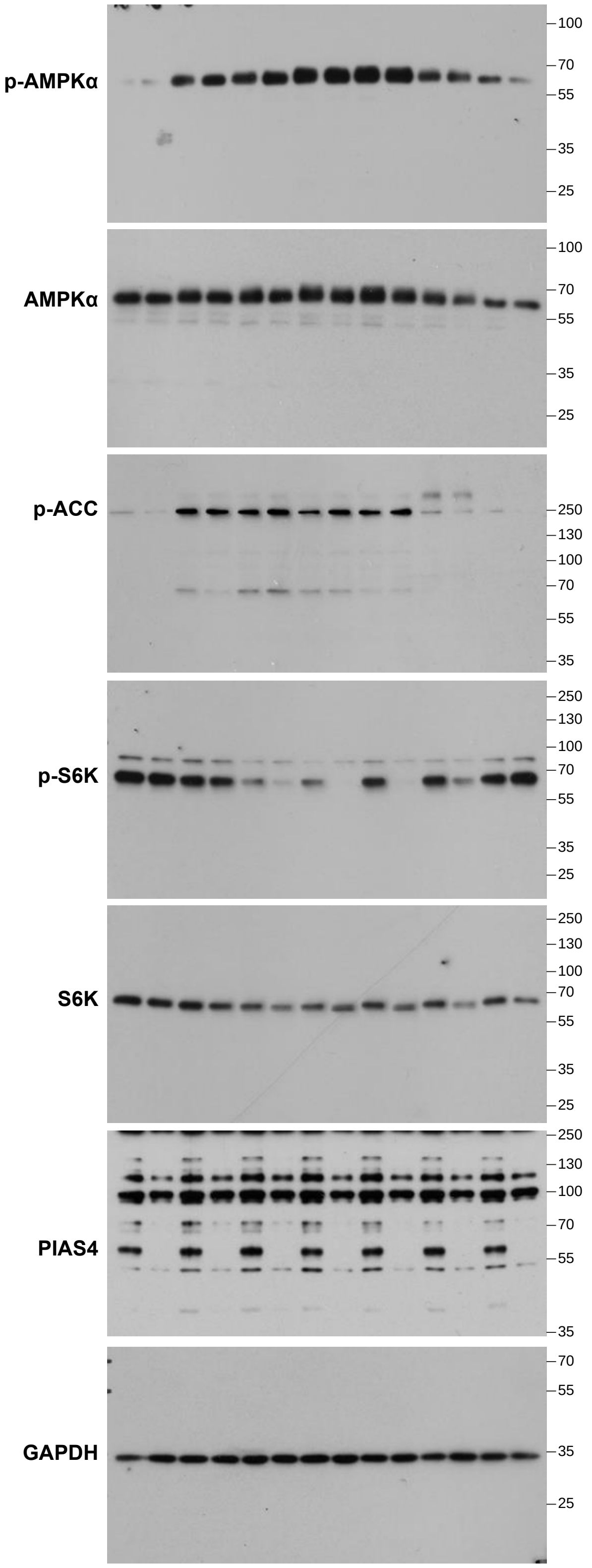

Supplementary Figure 10. Uncropped blots for Figure 1b and Figure 1c

Supplementary Figure-11 (Makela)

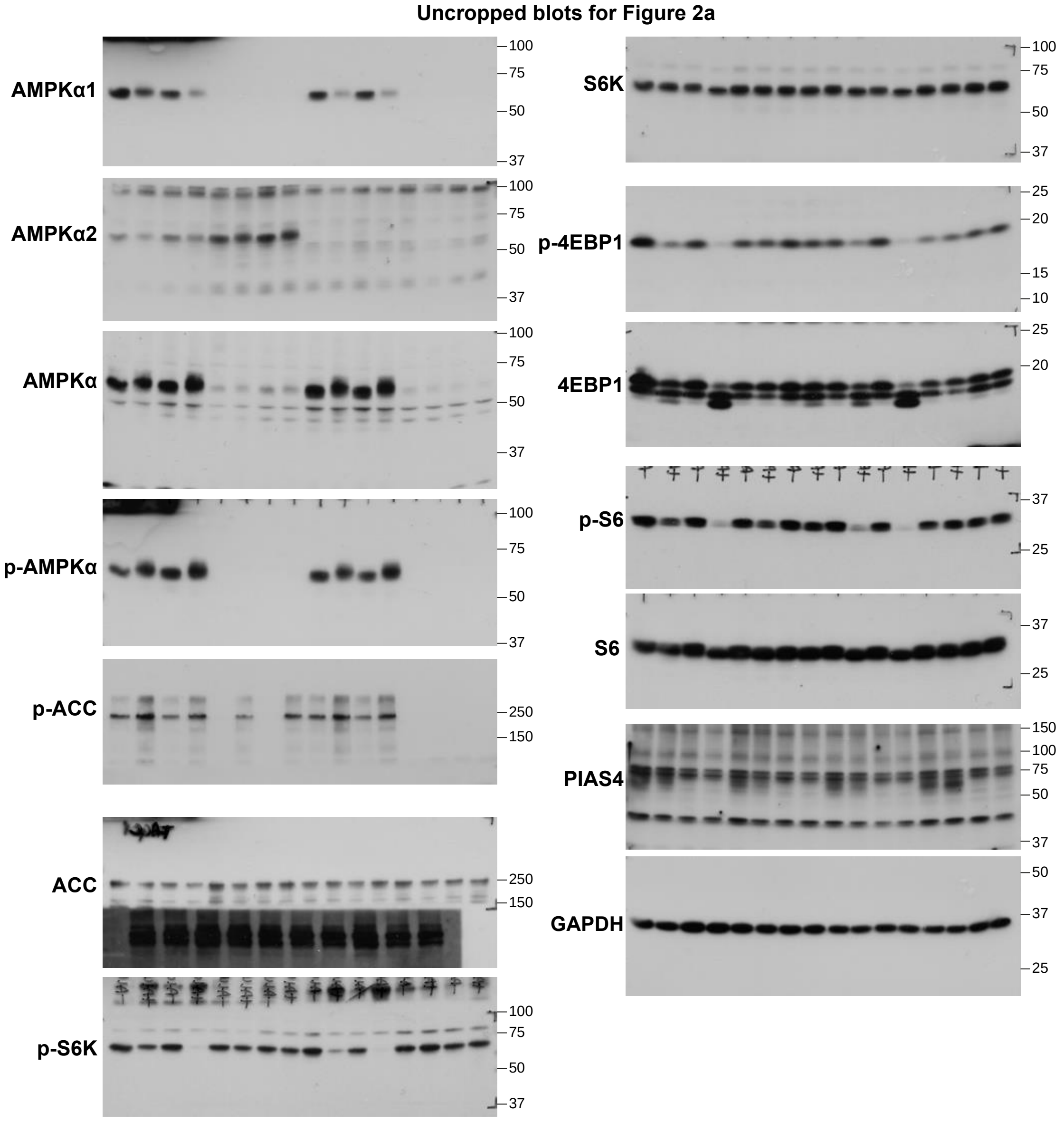

Supplementary Figure 11. Uncropped blots for Figure 2a

Supplementary Figure-12 (Makela)

Uncropped blots for Figure 2b

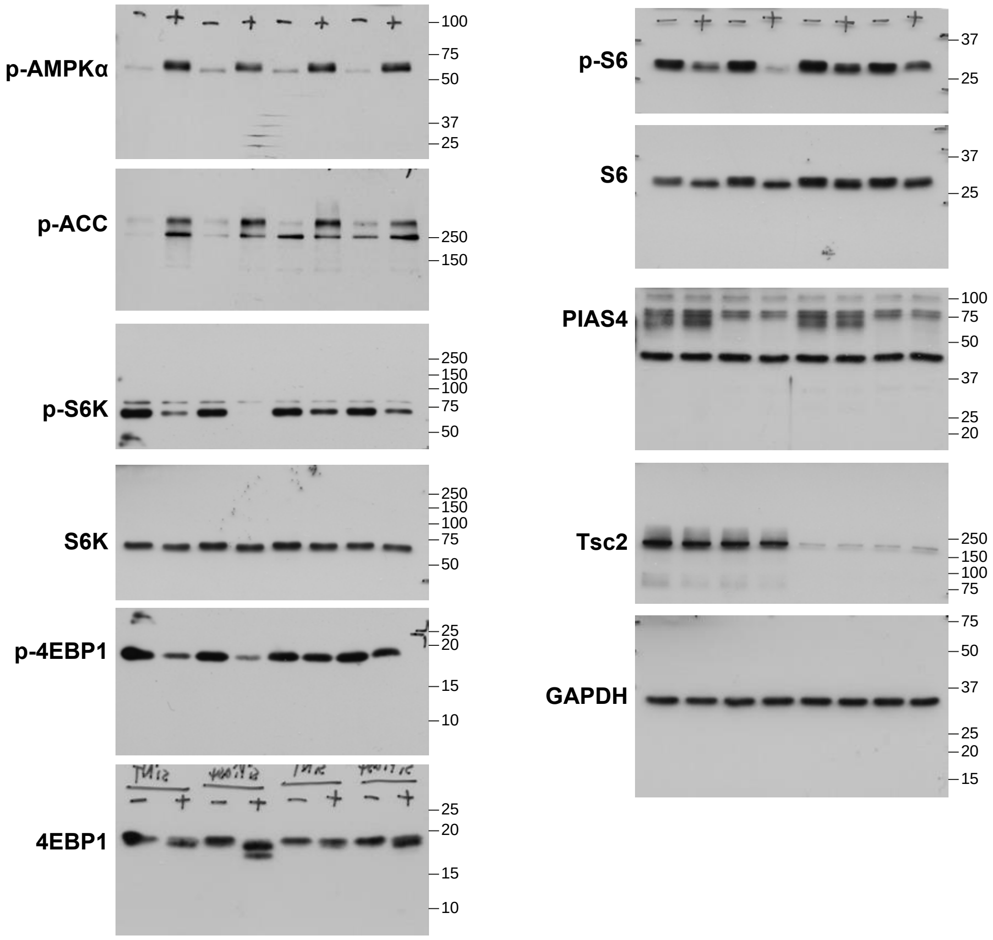

Uncropped blots for Figure 2c

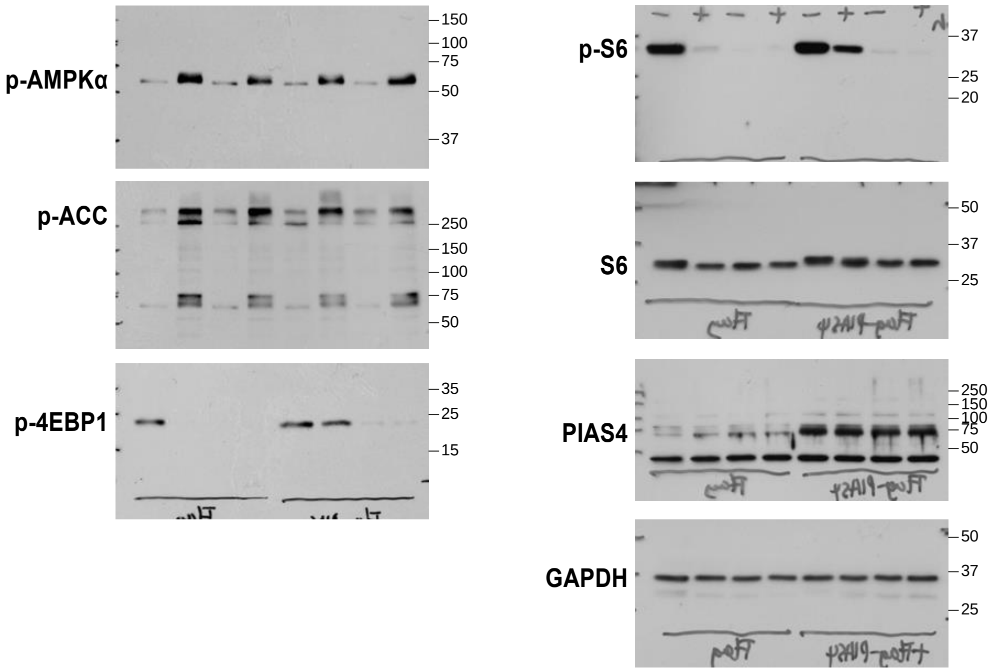

Supplementary Figure 12. Uncropped blots for Figure 2b and Figure 2c

Supplementary Figure-13 (Makela)

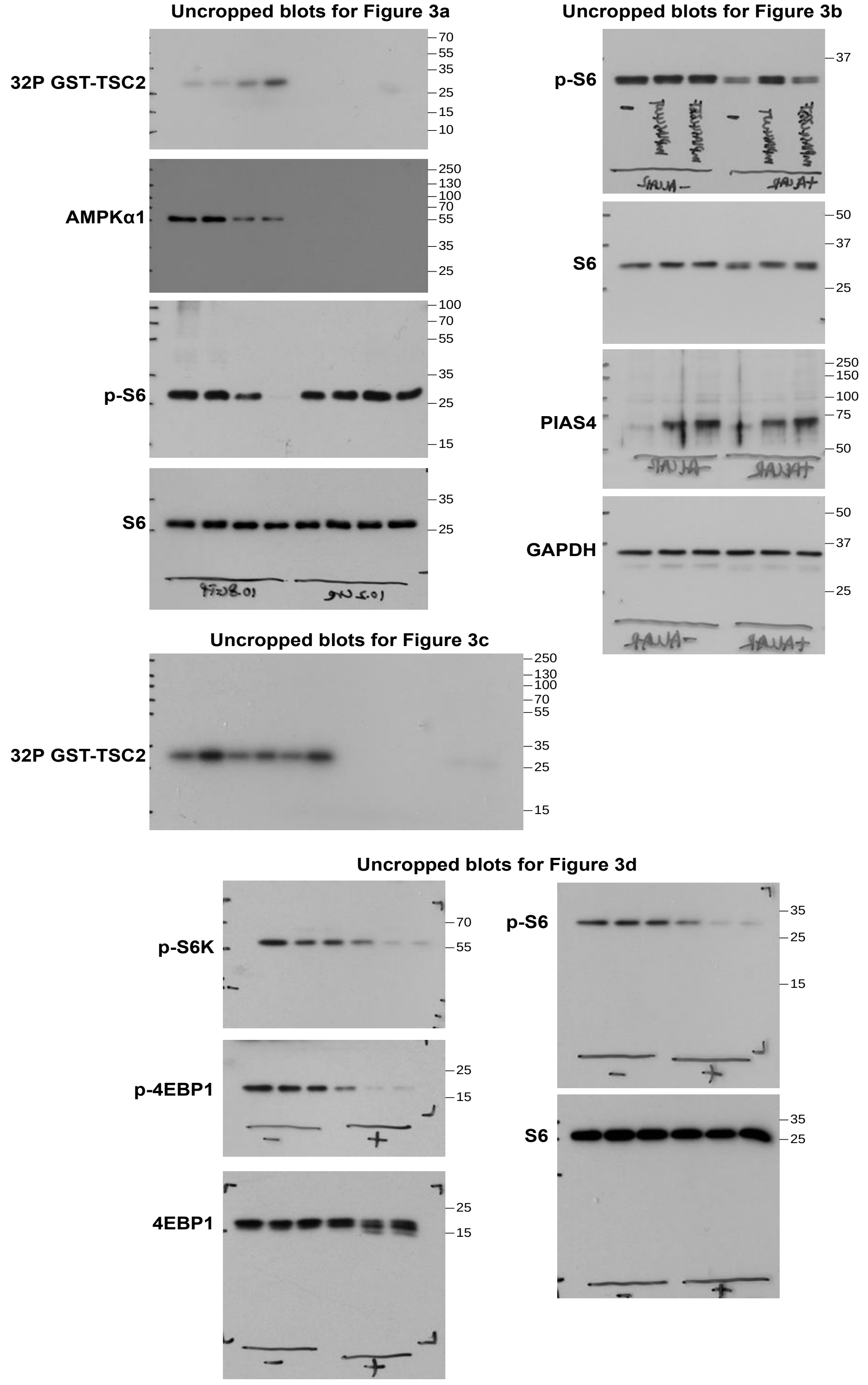

Supplementary Figure 13. Uncropped blots for Figure 3a, Figure 3b, Figure 3c, and Figure 3d

Supplementary Figure-14 (Makela)

Uncropped blots for Figure 4e

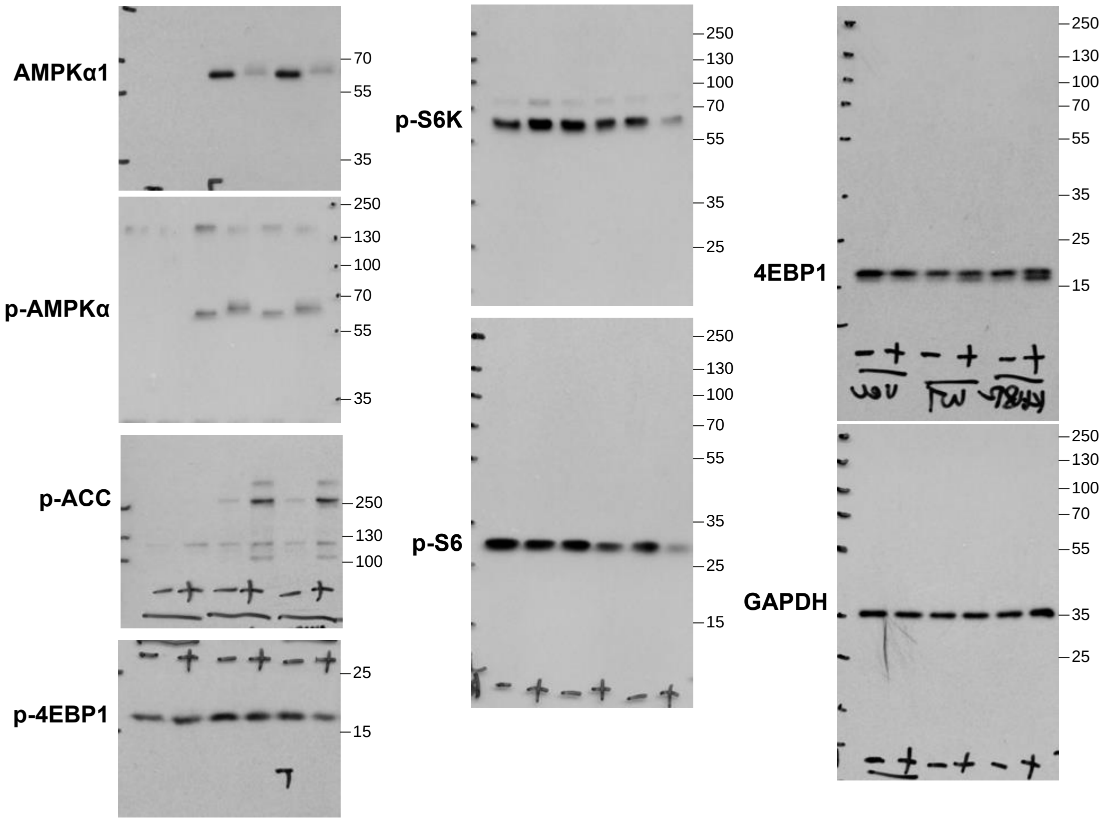

Uncropped blots for Figure 5a

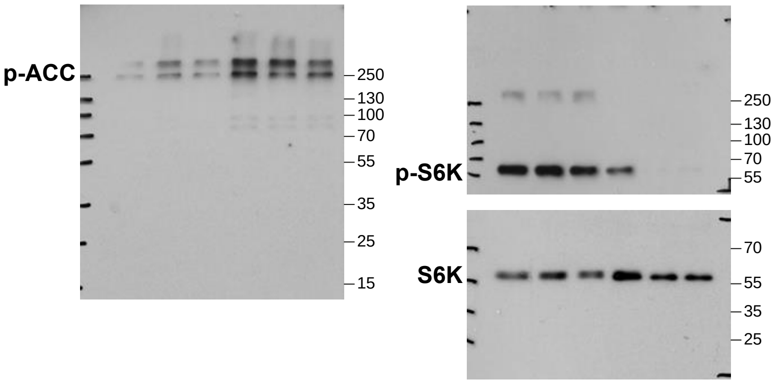

Supplementary Figure 14. Uncropped blots for Figure 4e and Figure 5a

Supplementary Table 1: Clones identified in yeast two-hybrid screens using either AMPKα1 or AMPKα2 as baits.

Bait	Library	Gene	Accession No.	Clones
AMPKα1	Liver	RILP	NM_031430	4
AMPKα1	Liver	TRIP6	NM_003302	5
AMPKα1	Liver	DISC1	NM_018662	1
AMPKα1	Liver	C3	NM_000064	1
AMPKα1	Liver	SF4	NM_172231	1
AMPKα1	Liver	SDHB	NM_003000	1
AMPKα1	Liver	YLPM1	XM_940570	1
AMPKα1	Liver	CASP8AP2	NM_012115	1
AMPKα1	Brain	DEAF1	NM_021008	21
AMPKα1	Brain	EWSR1	NM_005243	1
AMPKα1	Brain	C1orf 165	NM_024603	2
AMPKα1	Brain	OS9	NM_006812	1
AMPKα2	Liver	PIAS3	NM_006099	5
AMPKα2	Liver	PPP1R12C	NM_017607	3
AMPKα2	Liver	TRIP6	NM_003302	7
AMPKα2	Liver	SMN1	NM_000344	2
AMPKα2	Liver	DPYS	NM_001385	1
AMPKα2	Liver	PDLIM5	NM_006457	2
AMPKα2	Liver	HNRPK	BC014980	1
AMPKα2	Liver	CTAGE-5B	AF338234	1
AMPKα2	Liver	CFB	BC007990	1

AMPKα2	Liver	CPS1	XM_001146466	1
AMPKα2	Liver	UBB	NM_018955	1
AMPKα2	Liver	PHC2	BC068573	1
AMPKα2	Liver	PRKCBP1	HS569M23	1
AMPKα2	Liver	AADAT	NM_182662	1
AMPKα2	Liver	TACC2	BC015736	1
AMPKα2	Brain	ZBED	NM_032367	5
AMPKα2	Brain	PPP1R7	NM_002712	1
AMPKα2	Brain	KHDRBS1	BC019109	2
AMPKα2	Brain	PRKAG1	XM_509039	1
AMPKα2	Brain	BRUNOL4	XM_001128998	2
AMPKα2	Brain	PHC2	BC068573	3
AMPKα2	Brain	HNRPK	BC014980	1
AMPKα2	Brain	EIF4A1	NM_001416	1

Supplementary Table 2: Primers used in mutagenesis

Primer name	Sequence
PIAS4_C335F_F	CCGTGCAGAGACCTTCGCACACCTGC
PIAS4_C335F_R	GCAGGTGTGCGAAGGTCTCTGCACGG
AMPKα1_K15R_F	GTCGTGTTTCTGCCTCTCGGCTGTCGCCATC
AMPKα1_K15R_R	GATGGCGACAGCCGAGAGGCAGAAACACGAC
AMPKα1_K17R_F	CCCGTCGTGTCTCTGCTTCTCGGCTGTCGC
AMPKα1_K17R_R	GCGACAGCCGAGAAGCAGAGACACGACGGG
AMPKα1_K23R_F	GACGGGCGGGTGAGGATCGGCCACTAC
AMPKα1_K23R_R	GTAGTGGCCGATCCTCACCCGCCCGTC
AMPKα1_K40R_F	GCCAACCTTCACTCTGCCGAAGGTGCCGA

AMPKα1_K40R_R	TCGGCACCTTCGGCAGAGTGAAGGTTGGC
AMPKα1_K42R_F	CGGCACCTTCGGCAAAGTGAGGGTTGGCAAAC
AMPKα1_K42R_R	GTTTGCCAACCCTCACTTTGCCGAAGGTGCCG
AMPKα1_K45R_F	CCCAGTCAATTCATGTCTGCCAACCTTCACTTTGCC
AMPKα1_K45R_R	GGCAAAGTGAAGGTTGGCAGACATGAATTGACTGGG
AMPKα1_K52R_F	CTTCACAGCTACTCTATGCCCAGTCAATTCATGTTTGC
AMPKα1_K52R_R	GCAAACATGAATTGACTGGGCATAGAGTAGCTGTGAAG
AMPKα1_K56R_F	CTTCTGTCGATTGAGTATCCTCACAGCTACTTTATGCCC
AMPKα1_K56R_R	GGGCATAAAGTAGCTGTGAGGATACTCAATCGACAGAAG
AMPKα1_K62R_F	CATCAAGGCTCCGAATCCTCTGTCGATTGAGTATC
AMPKα1_K62R_R	GATACTCAATCGACAGAGGATTCGGAGCCTTGATG
AMPKα1_K71R_F	TGAATTTCTCTGCGGATTCTTCCTACCACATCAAGGC
AMPKα1_K71R_R	GCCTTGATGTGGTAGGAAGAATCCGCAGAGAAATTCA
AMPKα1_K80R_F	ATGCCTGAAAAGCCTGAGGTTCTGAATTTCTCTGCG
AMPKα1_K80R_R	CGCAGAGAAATTCAGAACCTCAGGCTTTTCAGGCAT
AMPKα1_K89R_F	AGCTTTTCAGGCATCCTCATATAATTAGACTGTACCAGGTCA
AMPKα1_K89R_R	TGACCTGGTACAGTCTAATTATATGAGGATGCCTGAAAAGCT
AMPKα1_K118R_F	TTCATCCAGCCTTCCATTCCTACAGATATAATCAAATAGCTCTCC
AMPKα1_K118R_R	GGAGAGCTATTTGATTATATCTGTAGGAATGGAAGGCTGGATGAA
AMPKα1_K125R_F	CGCCGACTTTCTCTTTCATCCAGCCTTCCATTCTTA

AMPKα1_K125R_R	TAAGAATGGAAGGCTGGATGAAAGAGAAAGTCGGCG
AMPKα1_K152R_F	CATATGGTGGTCCATAGAGATTTGAGACCTGAAAATGTCCTG
AMPKα1_K152R_R	CAGGACATTTTCAGGTCTCAAATCTCTATGGACCACCATATG
AMPKα1_K165R_F	GAAAGACCAAAATCAGCTATCCTTGCATTCATGTGTGCATCAA
AMPKα1_K165R_R	TTGATGCACACATGAATGCAAGGATAGCTGATTTTGGTCTTTC
AMPKα1_K235R_F	GATCCCATCACATATCTTCCTAAAAAGAGTTGGCACATGGTC
AMPKα1_K235R_R	GACCATGTGCCAACTCTTTTTAGGAAGATATGTGATGGGATC
AMPKα1_K236R_F	GAAGATCCCATCACATATCCTCTTAAAAAGAGTTGGCACATGG
AMPKα1_K236R_R	CCATGTGCCAACTCTTTTTAAGAGGATATGTGATGGGATCTTC
AMPKα1_K257R_F	CACCTGCAGCATATGTCTCAAAAGGCTAATCACAGAAGG
AMPKα1_K257R_R	CCTTCTGTGATTAGCCTTTTGAGACATATGCTGCAGGTG
AMPKα1_K266R_F	TTTGATTGTGGCCCTCCTCATGGGATCCACCTG
AMPKα1_K266R_R	CAGGTGGATCCCATGAGGAGGGCCACAATCAAA
AMPKα1_K271R_F	TGTTCCCTGATATCTCTGATTGTGGCCCTCTTCATG
AMPKα1_K271R_R	CATGAAGAGGGCCACAATCAGAGATATCAGGGAACA
AMPKα1_K280R_F	GATATCAGGGAACATGAATGGTTTAGACAGGACCTTCCA
AMPKα1_K280R_R	TGGAAGGTCCTGTCTAAACCATTCATGTTCCCTGATATC
AMPKα1_K285R_F	GATCCTCAGGAAAGAGATATCTTGGAAGGTCCTGTTTAAAC

AMPKα1_K285R_R	GTTTAAACAGGACCTTCCAAGATATCTCTTTCCTG AGGATC
AMPKα1_K305R_F	TCAAACTTTTCACATACTTCTCTTAAGGCTTCATCA TCAATCATGG
AMPKα1_K305R_R	CCATGATTGATGATGAAGCCTTAAGAGAAGTATGT GAAAAGTTTGA
AMPKα1_K310R_F	TCCTCTTCTGAGCACTCAAACCTTTCACATACTTCT TTTAAGG
AMPKα1_K310R_R	CCTTAAAAGAAGTATGTGAAAGGTTTGAGTGCTCA GAAGAGGA
AMPKα1_K349R_F	GTCGCCAAATAGAAATCTCTGGCTTCATTCATTAT TCTCCTG
AMPKα1_K349R_R	CAGGAGAATAATGAATGAAGCCAGAGATTTCTATT TGGCGAC
AMPKα1_K396R_F	CCTTACACCTTGGTGTTTGGATCTCTGTGGATTTAA TTCATCAAG
AMPKα1_K396R_R	CTTGATGAATTAAATCCACAGAGATCCAAACACCA AGGTGTAAGG
AMPKα1_K398R_F	CTTTCCTTACACCTTGGTGTCTGGATTTCTGTGGAT TTAATTCATC
AMPKα1_K398R_R	GATGAATTAAATCCACAGAAATCCAGACACCAAG GTGTAAGGAAAG
AMPKα1_K404R_F	CTAAATGCCATTTTGCTCTCCTTACACCTTGGTGTT TGGATT
AMPKα1_K404R_R	AATCCAAACACCAAGGTGTAAGGAGAGCAAAATG GCATTTAG
AMPKα1_K406R_F	TTCCTAAATGCCATCTTGCTTTCCTTACACCTTGGT GTTTG
AMPKα1_K406R_R	CAAACACCAAGGTGTAAGGAAAGCAAGATGGCAT TTAGGAA
AMPKα1_K429R_F	CCATTCATAATCCAATTGTCTGATTGCTCTACATAC TTCTGCCA
AMPKα1_K429R_R	TGGCAGAAGTATGTAGAGCAATCAGACAATTGGA

	TTATGAATGG
AMPKα1_K436R_F	GCAAATAATATGGGTTTACAACCCTCCATTCATAA TCCAATTGTTTGATT
AMPKα1_K436R_R	AATCAAACAATTGGATTATGAATGGAGGGTTGTAA ACCCATATTATTTGC
AMPKα1_K436R_F	GCAAATAATATGGGTTTACAACCCTCCATTCATAA TCCAATTGTTTGATT
AMPKα1_K436R_R	AATCAAACAATTGGATTATGAATGGAGGGTTGTAA ACCCATATTATTTGC
AMPKα1_K448R_F	TGCTTGTCACAGGATTCCTCCTTCGTACACGCAAA
AMPKα1_K448R_R	TTTGCGTGTACGAAGGAGGAATCCTGTGACAAGCA
AMPKα1_K485R_F	GGAGTAGCAGTCCCTGATCTGGCTTCTGTAATTTC AT
AMPKα1_K485R_R	ATGAAATTACAGAAGCCAGATCAGGGACTGCTAC TCC
AMPKα1_K515R_F	ACTTCTGAGGATCTTCCTTGAGCCTCAGCATCTGA
AMPKα1_K515R_R	TCAGATGCTGAGGCTCAAGGAAGATCCTCAGAAG T
AMPKα1_K555R_F	GGTTCTATTGTGCAAGAATTCTAATTAGATTTGCA CACATCTCAAAAAATTCTATTG
AMPKα1_K555R_R	CAATAGAATTTTTTGAGATGTGTGCAAATCTAATT AGAATTCTTGCACAATAGAACC

Supplementary Table 3: Dharmacon siRNAs used in this study

siRNA name	siRNA ID	Targeting gene
siNT pool	D-001810-10	
siPias1 pool	L-059344-01	Mouse *Pias1*
siPias2 pool	L-055561-01	Mouse *Pias2*
siPias3 pool	L-045382-00	Mouse *Pias3*

siPias4 pool	L-048649-01	Mouse *Pias4*
siTsc2 pool	L-047050-00	Mouse *Tsc2*
siPias4-10	J-048649-10	Mouse *Pias4*
siPias4-11	J-048649-11	Mouse *Pias4*
siUbc9-1	D-040661-01	Mouse *Ubc9*
siUbc9-2	D-040661-02	Mouse *Ubc9*
siPIAS4 pool	L-006445-00	Human *PIAS4*
siUBC9 pool	L-004910-00	Human *UBC9*

Supplementary Table 4: Primers used in Real-time qPCR:

**Primer name**	**Sequence**
Mouse_*Pias1*_F	CGAGCAAAGGGAATAAGGAA
Mouse_*Pias1*_R	TGAAGGTGGGAGCAGGTAAG
Mouse_*Piasxα*_F	GTAAGGGTGCCCAGTGTGAC
Mouse_*Piasxα*_R	TCAGAAGATGCTCCAAGCTG
Mouse_*Piasxß*_F	GATCCCCAGTACTGTCCTCCT
Mouse_*Piasxß*_R	CTGCTGGTTATGACCCCTGT
Mouse_*Pias3*_F	GGACGTGTCCTGTGTGTGAC
Mouse_*Pias3*_R	CTCTGATGCCTCCTTCTTGG
Mouse_*Pias4*_F	GGAGGCCAAAAACATGGTGAT
Mouse_*Pias4*_R	GGGCTACAGTCGAACTGCAC
Mouse_*Gapdh*_F	TGTTCCTACCCCCAATGTGT
Mouse_*Gapdh*_R	TGTGAGGGAGA TGCTCAGTG

Human_*PIAS4*_F	GGTGAAGCTGCCGTTCTTTA
Human_*PIAS4*_R	TCTCGTTGTTCTGTGGGACTAA
Human_*GAPDH*_F	CGACCACTTTGTCAAGCTCA
Human_*GAPDH*_R	TGTGAGGGAGA TGCTCAGTG
